# Reactions of Tetracyanoethylene with Aliphatic and Aromatic Amines and Hydrazines and Chemical Transformations of Tetracyanoethylene Derivatives

**DOI:** 10.3390/molecules29194727

**Published:** 2024-10-06

**Authors:** Elizaveta Ivanova, Margarita Osipova, Yhtyyar Kadyrov, Sergey Karpov, Svetlana Markova, Ekaterina Zazhivihina, Lubov Umanova, Tatyana Vasilieva, Yurii Mitrasov, Yulia Smolkina, Oleg Nasakin

**Affiliations:** 1 Organic and Pharmaceutical Chemistry Department, Ulyanov Chuvash State University, Moskovsky Prospect, 15, 428015 Cheboksary, Russia; lizachimic@mail.ru (E.I.); margolev1966@icloud.com (M.O.); kadyrow_1506@mail.ru (Y.K.); serg31.chem@mail.ru (S.K.); konxmark@mail.ru (S.M.); eiz2020@mail.ru (E.Z.); luba.yumanova@mail.ru (L.U.); tava52@mail.ru (T.V.); smolka7979@mail.ru (Y.S.); 2 Department of Scientific Chemistry Education, Yakovlev Chuvash State Pedagogical University, K. Marx Street, 38, 428000 Cheboksary, Russia

**Keywords:** tetracyanoethylene, tricyanovinylations, aromatic amines, Schiff bases, benzamidines, tricyanovinyl dyes, dicarbonitriles, butadiene-1,1,2,2-tetracarbonitriles, aryl-quinazolines

## Abstract

The significant synthetic potential and reactivity of tetracyanoethylene (TCNE) have captured the interest of numerous chemical communities. One of the most promising, readily achievable, yet least explored pathways for the reactivity of TCNE involves its interaction with arylamines. Typically, the reaction proceeds via tricyanovinylation (TCV); however, deviations from the standard chemical process have been observed in some instances. These include the formation of heterocyclic structures through tricyanovinyl intermediates, aliphatic dicarbonitriles through the cleavage of the C–C bond of a tetracyanoethyl substituent, complexation, and various pericyclic reactions. Therefore, the objective of this study is to review the diverse modes of interaction of TCNE with aromatic nitrogen-containing compounds and to focus the attention of the chemical community on the synthetic capabilities of this reagent, as well as the various biological and optical activities of the structures synthesized based on TCNE.

## 1. Introduction

TCNE is a promising synthon in organic chemistry, comprising colorless crystals that sublimate at 120 °C and melt at 198–200 °C in a closed capillary [1]. The best yield (85–89%) [2] is achieved by heating brommalonitrile with copper (2:1). It has been 70 years since its discovery, and 65 years since the beginning of its study by Dupont chemists in 1958 [1]. Since then, tens of thousands of articles and reviews have been published on the remarkable synthetic activity of this unique compound. One of the simplest, yet most-promising and least-studied areas of its chemistry is the “tricyanovinylation” of aromatic amines and its potential capabilities. We have compiled all the literature up to 2022 on this subject to draw the attention of the chemical community to its preparative potential and the various types of biological and light-absorbing activities of the carbonitrile organic compounds obtained from it.

Preparatively, highly cost-effective, high-yield syntheses based on tetracyanoethylene (TCNE) and aromatic amines involve tricyanovinylation reactions (TCV). TCV with primary and secondary arylamines presupposes the formation of a tricyanovinyl substituent on the amino group, while with tertiary arylamines, it occurs in pairs with respect to the substituent. These reactions typically proceed smoothly, with high-speed and yield-of-target compounds. TCV yields washing-resistant dyes suitable for hydrophobic fibers [3], components for photo- and electroluminescent devices [4], conductive organic materials [5,6] applicable for TV transmission and recording [6,7], as well as solar batteries [8]. Tricyanovinyl derivatives of aniline also exhibit various types of biological activity, including efficacy against Parkinson’s disease [9], antiviral [9], fungicidal [9,10], antimycotic [9], herbicidal [9], antibacterial [9,10], anthelmintic [10], and antifungal [10,11] activities. Furthermore, nanostructured colored optical films with enhanced electrical conductivity under UV radiation [12] have been developed based on a tricyanovinylated diethylaniline derivative.

Tricyanilamines are also of interest in medicine. They inhibit oxidative phosphorylation even at a concentration of 10^−7^ mol^−1^ and simultaneously reduce the amount of glutathione, a peptide responsible for detoxifying xenobiotics, regulating redox processes in the cell, immune function, and the oxidative state of important sulfhydryl protein groups by up to 30% [13], as confirmed by reaction with thiols [14]. Derivatives of TCNE and arylamines also serve as ideal synthons for the synthesis of biologically active heterocycles. For example, through interaction with arylbenzamidines [15] and 2-aminobenzylamine [16], they can be used in the synthesis of quinazolines [17], which act as kinase inhibitors, promoting the division of cancer cells [18]. However, in some cases, the reaction proceeds differently.

## 2. Unusual Chemical Reactions with TCNE

**Scheme 1:**
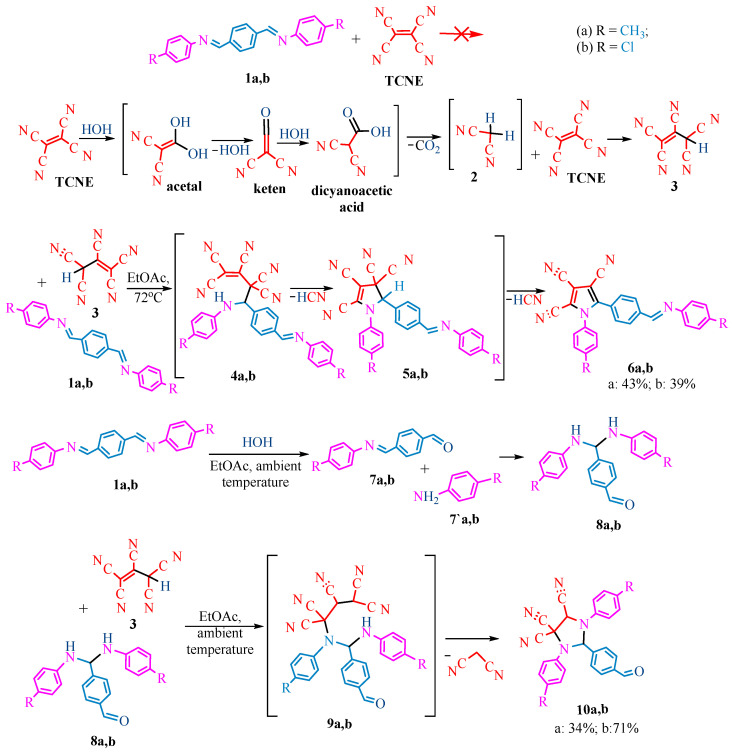
Reaction of **TCNE** with bis-airlmetanimines. Aniline fragments are highlighted in pink color, terephtalic aldehyde—in blue color, TCNE—in red color, water—in turquoise color. Black bonds in the compounds indicate the addition of one molecule to the other, as well as addition of functional groups within the molecule.

**Scheme 2:**
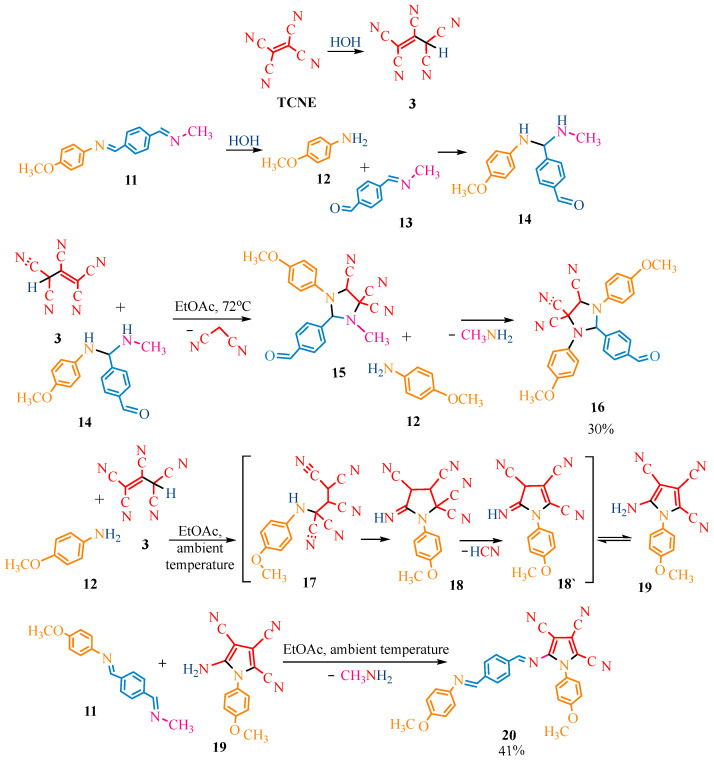
Reaction of **TCNE** with methyl-methoxy phenyl methanimine. Aniline derivative is market in orange color, terephtalic aldehyde—in blue color, methylamine—in crimson color, TCNE in red color. Black bonds in the compounds indicate the addition of one molecule to the other, as well as addition of functional groups within the molecule.

**Scheme 3:**
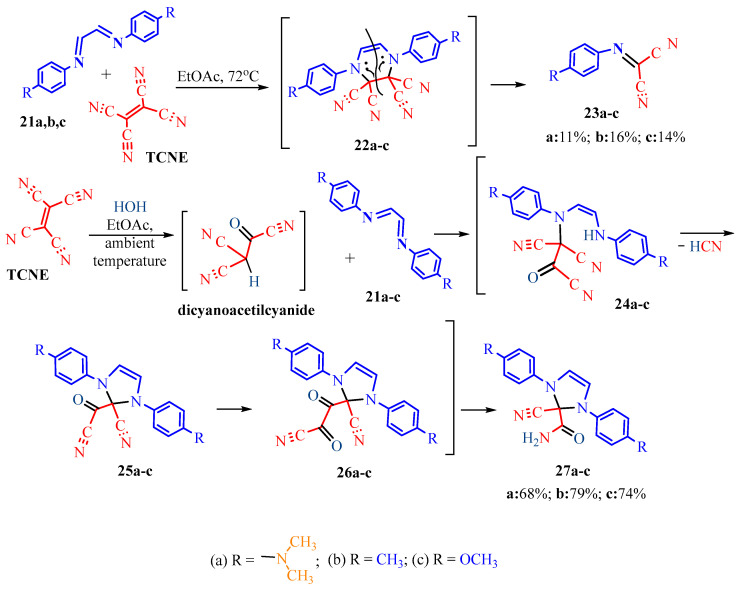
Reaction of **TCNE** with diarylethanediimines. Aniline derivative is market in orange color, terephtalic aldehyde—in blue color, methylamine—in crimson color. Black bonds in the compounds indicate the addition of one molecule to the other, as well as addition of functional groups within the molecule.

The publication [19] presents interesting reactions of **TCNE** with nitrogen-containing compounds. The authors conducted **TCNE** reactions with Schiff bases in ethyl acetate at 72 °C and at room temperature (Scheme 1, Scheme 2 and Scheme 3), leading to different results. For instance, the interaction of **TCNE** with terephthalaldehyde derivatives **1a**,**b** at high temperature (72 °C) resulted in pyrroles **6a**,**b**, whereas at room temperature, it led to imidazolidines **10a**,**b**. Similarly, the interaction with Schiff’s base **11** at high temperature resulted in imidazolidine **16**, while at room temperature, **11** cyclized into pyrrole **20**. In the case of glyoxal Schiff’s bases **21a**–**c**, at 72 °C, arylcarbonimidoyldicyanides **23a**–**c** are formed, whereas at 20 °C, dihydroimidazolecarboxamides **27a**–**c** are obtained.

Given the availability of the publication in the public domain, we propose our own schemes showing the formation of reaction products in Scheme 1, Scheme 2 and Scheme 3. We proposed that in several cases (Scheme 1, Scheme 2 and Scheme 3), **TCNE** was hydrolyzed in ethyl acetate containing up to 8% water prior to reaction with Schiff’s bases. This hydrolysis consequently led to **acetal**, **keten**, **dicyanoacetic acid** decarboxylated to malonnitrile **2**, which was finally added to **TCNE** to give pentacyanopropene **3** [20]. Scheme 1 shows the addition of pentacyanopropene **3** to the C=N bond of imines **1a**,**b** by heating at 72 °C. The formed Michael adducts **4a**,**b** underwent intramolecular cyclysis to give dihydropyrroles **5a**,**b**, which were converted to pyrroles **6a**,**b** after prussic acid elimination in yields of 43% and 39%, respectively. Meanwhile, we propose that the original compounds **1a**,**b** were hydrolyzed at room temperature to aldehydes **7a**,**b** and amines **7′a**,**b**. The reaction between them (**1a**,**b** and **7a**,**b**) occurred as a Michael-type addition leading to bis(arylamino)methylarylaldehydes **8a**,**b**. The hydrolysis products **8a**,**b**, via intermediates **9a**,**b**, finally formed diarylimidazolidine-tricarbonitriles **10a**,**b** in yields of 34% and 71%, respectively (Scheme 1).

The reaction of **TCNE** with the methyl derivative of methanimine **11** (Scheme 2) differs from the previous scheme (Scheme 1). Tetracyanoethylene was converted to penthacyanopropene **3**, following a similar pattern to the reaction described previously. For products **16** and **20** (Scheme 2), we assumed that Schiff’s base **11** was hydrolyzed to amine **12** and aldehyde **13** at both temperatures (72 °C and ambient). This hydrolysis at 72 °C facilitated the formation of bis(arylamino)methylarylaldehyde **14**, and its reaction with penthacyanopropene **3** led to imidazolidine **15** by analogy with Scheme 1 (similar transformations of the original compounds **1a**,**b** and **TCNE** took place at ambient temperature). The *N*-methyl of imidazolidine **15** was presumably replaced by residual amino-anisole 12, which is the product of the hydrolysis of Schiff’s base **11**, yielding 30% of the final product 16. At room temperature, Schiff’s base **11** underwent partial hydrolysis to amine **12**, which probably reacted with pentacyanopropenide **3** to give pentacyanopropane **17**. The latter underwent intramolecular cyclization into pyrazolone imine **18**. Subsequent elimination of prussic acid **18′** and tautomerization produced pyrrole **19**, which then interacted with the original compound **11**. The elimination of methylamine resulted in the final pyrrole **20** with a yield of 41% (Scheme 2).

Referring to Scheme 3, it was assumed that at 72 °C, **TCNE** interacts with diarylethanediimines **21a**–**c** via a [4+2] cycloaddition, followed by cleavage of the C=C bond of the adducts **22a**–**c** and acetylene elimination to give imindicarbonitriles **23a**–**c** in yields of 11%, 16%, and 14%, respectively. At room temperature, **TCNE** is expected to hydrolyze to **dicyanoacetyl cyanide**, which differs from previous Scheme 1 and Scheme 2. The hydrolysis product is likely to enter the reaction with the original compounds **21a**–**c** as a CH-acid, adding conjugated double bonds **21a**–**c**. Intermediates of 1,4-addition **24a**–**c** consequently underwent intramolecular cyclization to dihydroimidazole **25a**–**c**. Cyano groups of carbonyl cyanide moieties **25a**–**c** were presumably hydrolyzed to α-oxoamides **26a**–**c**. In the final step, we assumed the typical α-oxoacid libation of carbon monoxide to form amides **27a**–**c** in yields of 68%, 79%, and 74%, respectively (Scheme 3).

The tricyanovinylation of dimethylaniline (**DMA**) is of significant interest. The resulting dye is employed in photovoltaics [5] and as a material for thin films in television and video recording technologies [6] owing to its high electrical conductivity [5]. However, the reaction mechanism between **DMA** and tetracyanoethylene (**TCNE**), which yields a compound of practical importance, remains incompletely understood [21].

One of the initial challenges was detecting the light absorption of the π-complex formed between **DMA** and **TCNE** using UV–visible spectroscopy, particularly when the reaction was conducted in polar solvents. To decelerate the reaction, isotopic kinetic studies were performed. In this approach, deuterium, being heavier than hydrogen, slows its incorporation into the **TCNE** multiple bond, thereby extending the lifetime of the π-complex. Through this method, the presence of the **TCNE** complex was confirmed by observing its decay in dichloromethane [21].

**Scheme 4:**
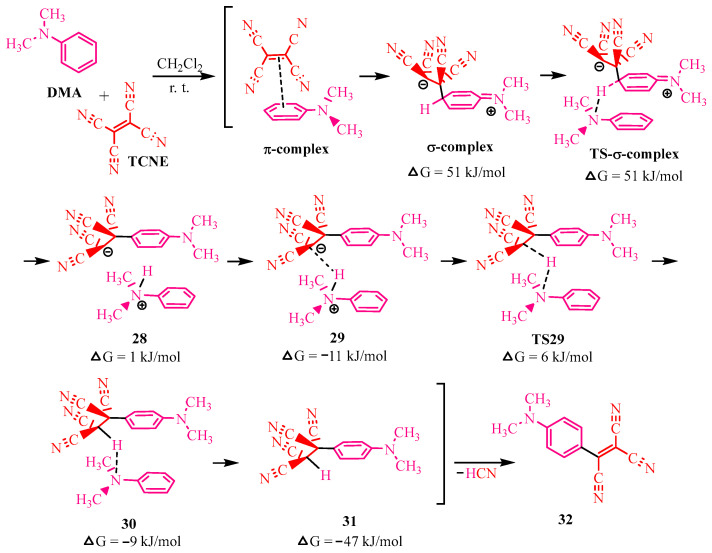
Mechanism of reaction of **TCNE** and **DMA.** Dimethylaniline is crimson, TCNE is red.

Further efforts were directed toward isolating the intermediates of the **DMA**-**TCNE** reaction. Synthesis in a nonpolar solvent—such as dioxane—at room temperature, resulted in the precipitation of the tetracyanoethyl derivative **31** (illustrated in Scheme 4). Compound **31** was recrystallized from benzene [21] and characterized using spectral methods. Based on the structure of compound **31**, the structure of its precursor, a σ-complex in the form of a zwitterion, was proposed (Scheme 4).

The authors of article [21], utilizing the available literature data, elucidated the intermediate interactions involved in the transformation of the σ-complex into tetracyanoethylated dimethylaniline 3 (Scheme 4). To achieve this, they employed the density functional theory (DFT) method. According to DFT, matter consists of interacting electrons within a lattice of atomic nuclei. The reaction was conducted in dichloromethane at room temperature under conditions identical to those used for the isotopic kinetic method [20].

By measuring electron density throughout the reaction and accounting for harmonic oscillations to capture transition states (**TS**), seven canonical structures were identified, and their free energies of formation (∆G, kJ/mol) were calculated (Scheme 4). **TCNE** initially attaches to **DMA**, forming a zwitterionic σ-complex via a π-complex intermediate. This zwitterion is then deprotonated by a second **DMA** molecule, yielding zwitterion **28** through the transition state (**TS-σ-complex**). The **DMA** cation, exhibiting acidic properties, donates a proton to the aromatic anion. Protonation of the tetracyanoethyl group occurs through the canonical structure **29**, the **TS29** transition state, and intermediate **30**. The final step involves the elimination of cyanohydrogen from tetracyanoethyl dimethylaniline **31**, resulting in the formation of tricyanovinyl **32**.

The authors of article [22] successfully improved the yield of tricyanovinyl dimethylaniline and identified the most environmentally friendly and economically viable method for its synthesis. The tricyanovinyl derivatives of aniline (**34a**–**h**) and 2-methylindole (**36i**–**l**) were synthesized using enzyme catalysts and a deep eutectic solvent (**DES**). The selection of specific enzymatic catalysts, namely lipase [23] and protease [24], was likely due to their high selectivity, reaction rate enhancement, mild operating conditions, and the non-toxic nature of the protein structures.

Deep eutectic solvent (**DES**) [25] offers several advantages over many low-boiling organic solvents. Besides being non-toxic and non-volatile (facilitating product isolation), **DES** is non-flammable, biodegradable, safe, inexpensive, and capable of increasing reaction rates [22]. A key advantage, in addition to those mentioned, is the recoverability of both the enzyme catalysts and **DES**, preserving their activity for reuse [22].

The enzymes were isolated in pure form from commercially available cell lines. Lipase, responsible for fat hydrolysis [23], was derived from the bacterial strain *Pseudomonas* sp., while protease, which catalyzes the cleavage of peptide bonds in proteins [24], was obtained from a strain of *Bacillus subtilis*. **DES** [25] was prepared by mixing choline chloride (**ChCl**) and urea.

**Scheme 5:**
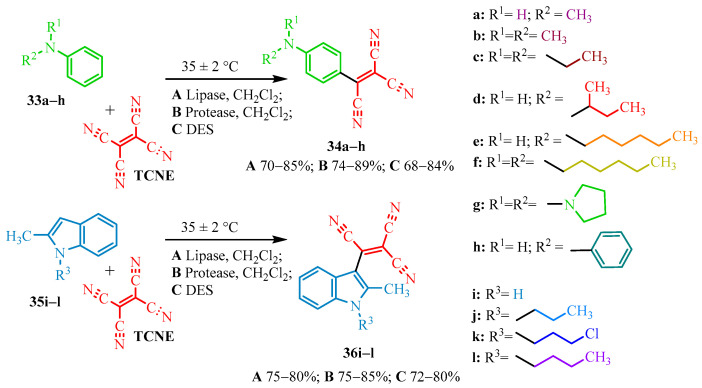
Synthesis of tricyanovinilic dyes from the derivatives of aniline and indole.

Three different methods were employed for the synthesis of **TCNE**-based tricyanovinyl derivatives of aniline (**34a**–**h**) and 2-methylindole (**36i**–**l**), all performed at 35 ± 2 °C, presumably to enhance the solubility of the protein catalysts and the melting of **DES**. The first method (**A**) utilized lipase in dichloromethane, while the second method (**B**) employed protease in the same solvent. Dichloromethane was likely selected due to its low boiling point and sufficiently high polarity, which helped accelerate the reaction and facilitate product isolation. The third method (**C**) used **DES**. Scheme 5 outlines the reactions between **TCNE** and anilines (**33a**–**h**) or 2-methylindoles (**35i**–**l**) under the corresponding reaction conditions, along with yield ranges for each methodology (**A**–**C**).

Table 1 presents the yields of the tricyanovinylated products along with the corresponding reaction times.

Additionally, the authors of article [22] investigated the reaction of methylaniline (**33a**) with **TCNE**. To optimize the yield of tricyanovinylmethylaniline and reduce the reaction time, various deep eutectic solvents (**DES**), conventional solvents, and solvent-free conditions were tested. The highest yield of 89% and the shortest reaction time of 5 min were achieved using a **DES** composed of choline chloride and urea. The results are summarized in Table 2.

**Figure 1:**
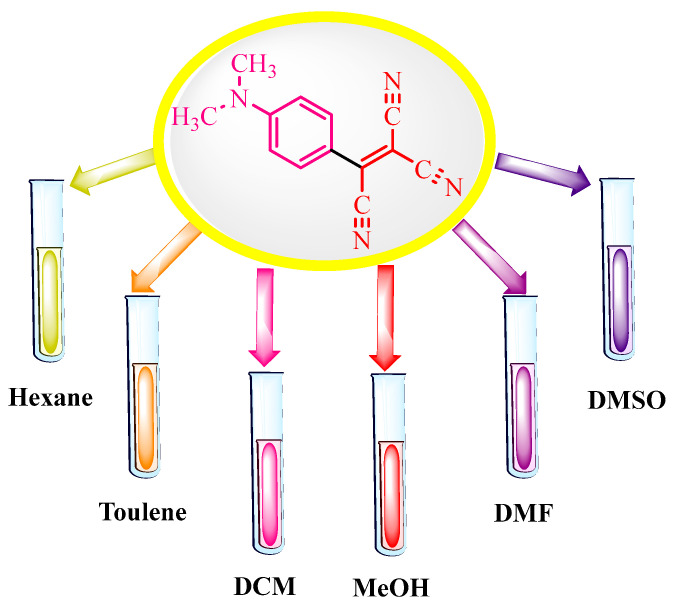
Tricyanovinilated dimethylaniline in daylight in various solvents.

The authors of [22] also investigated the influence of cyano groups on the optical properties of the synthesized compounds (see Table 9, “Absorption maxima for tricyanovinylated compounds”, in Section 6, “Molecular Research” and Figure 1, “Tricyanovinylated dimethylaniline in daylight in various solvents”, below).

**Scheme 6:**
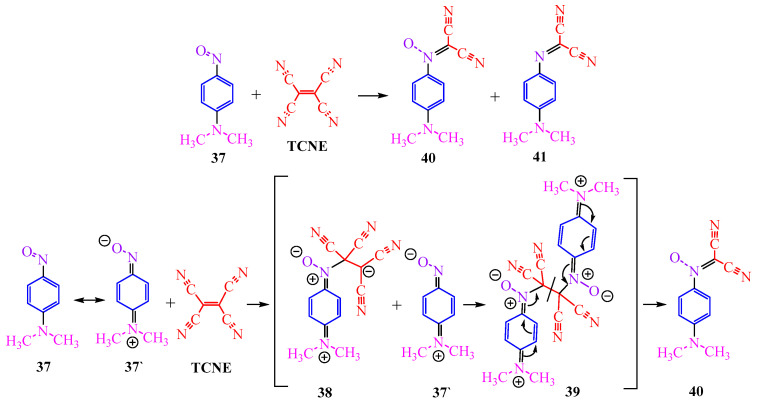
Reaction of **TCNE** with p-nitroso-*N*,*N*-dimethylaniline. Dimethilamine (pink) and nitroso-group (violet) in nitrosodimethylamine moiety are marked in individual colors, as they participate in the reaction.

The interaction of **TCNE** with p-nitrosodimethylaniline **37** is unusual (Scheme 6). The authors of publication [26] suggest the formation of compound **41** through the intermediate zwitterion **39**, with the attachment of a second molecule of arylamine **40**, followed by the cleavage of the double bond (Scheme 6).

The assumption regarding the intermediate zwitterion **39** is supported by the fact that the reaction with nitrobenzene **37** does not occur under similar conditions (i.e., mixing of reagents in DMF at 20 °C for 30 min) [26].

**Scheme 7:**
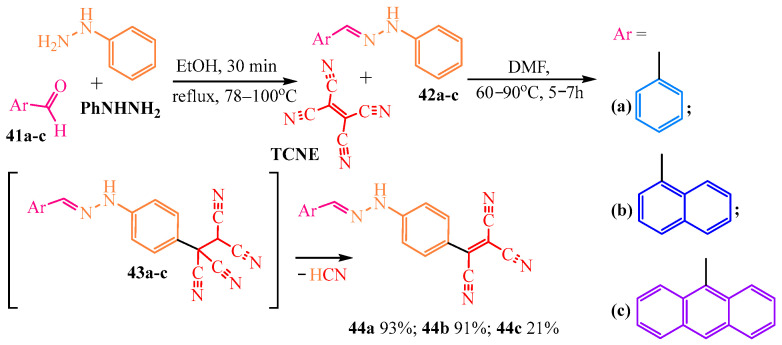
Reaction of **TCNE** with arylhydrazones.

The reaction of phenylhydrazones **42a**–**c** with **TCNE** takes an unusual course [8] (Scheme 7) in which the latter compound, rather than undergoing the typical proton substitution reaction at the nitrogen atom of the amino group **42a**–**c**, becomes incorporated at the para-position of the phenyl ring. The authors of [8] synthesized the target compounds **44a**–**c** in two stages: first, phenylhydrazine (**PhNHNH_2_**) and the corresponding arylaldehyde **41a**–**c** were refluxed in ethanol under 78–100 °C for 30 min to synthesize the hydrazones, and then, a tricyanovinyl radical was introduced through mixing Schiff’s bases **42a**–**c** with **TCNE** in DMF at 60–90 °C (Scheme 7).

**Scheme 8:**
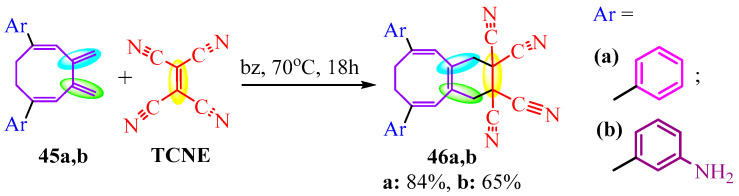
The [4+2] cycloaddition.

Aromatic amine **45b** reacts with the **TCNE** fragment of butadiene by Diels–Alder, instead of the reaction with amino group ([4+2]-cycloaddition, Scheme 8). In Scheme 8, the yield of aniline derivative **46b**, 65%, is provided for comparison with the phenyl derivative **46a**, 84% [27].

The authors of publication [28] investigated the capability of **TCNE** to form complexes with nitrogen- and oxygen-containing compounds, utilizing this property to establish spectroscopic characteristics that elucidate the behavior of drugs in the human body. The identification of **TCNE** complexes through spectroscopic methods enables the determination of the drug’s mechanism of action, the binding site of the active substance molecule to the biological target, and the physical and thermodynamic properties, allowing for the quantification of drug purity. Additionally, **TCNE**-based complex compounds exhibit activity against both Gram-positive and Gram-negative bacteria. The main advantage of the method proposed in article [28] is the ability to conduct studies without isolating the active substance from the medicinal compound, which traditionally involves lengthy processes and significant losses of the target product. In the experiment described in article [28], six drugs were utilized (for brief descriptions and molecular weights, see Section 6, “Molecular Research”).

**TCNE**-based complexes were synthesized by combining a medicinal substance in 20 mL of acetonitrile (resulting in a colorless solution) with **TCNE** in 20 mL of the same solvent. The reaction mixture was heated and stirred at 0.5 °C, followed by solvent evaporation, yielding a stable yellow complex. Figure 1 illustrates an example of complexation with sulfamethoxazole (***Drug 6***), where Solution 1 contains **TCNE**, Solution 2 contains ***Drug 6***, and Solution 3 represents the complex formed with **TCNE** after brief heating.

**Scheme 9:**
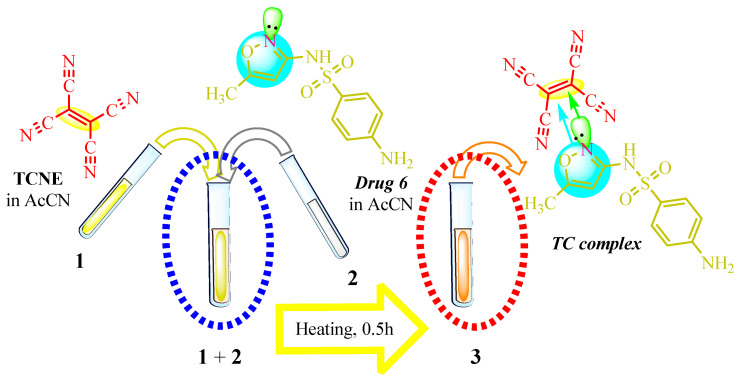
Color change in the solution with sulfomethoxazole. Colored circles highlight the centres of complexation between double TCNE bond (yellow), aromatic ring (blue) and nitrogen lone electrone pair (green).

Presumably, in this instance, the reaction of the TCV on the amino group did not occur for the following reasons: the use of an aprotic solvent, an insufficiently elevated temperature of the reaction mixture, solvent evaporation during the complexation stage, and the high electron density of the isoxazole ring (Scheme 9).

To analyze the obtained complex compounds, the authors of [28] conducted spectrophotometric measurements, stoichiometric titration, and determined the thermodynamic parameters of the compounds under investigation (see Table 11, “(A) Non-splitting and (B) splitting absorption of bonds of synthesized complexes”, in Section 6, “Molecular Research”). The authors of publication [28] also determined the thermodynamic parameters (refer to Table 10, “Thermodynamic parameters of complex compounds”, in Section 6, “Molecular Research”) and unveiled their correlations.

## 3. Heterocyclic Derivatives via Tricyanovinyl Intermediates

**Scheme 10:**
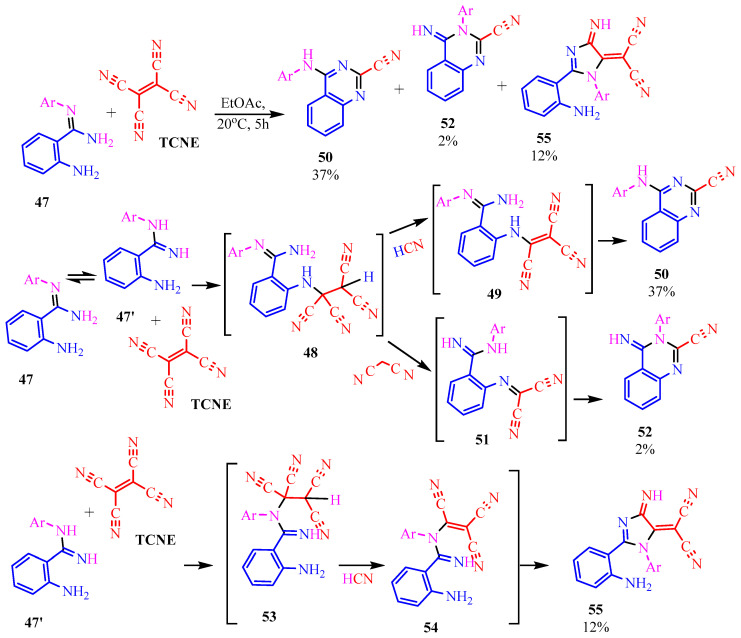
Interaction of **TCNE** with 2-amino-*N*-arylbenzamidine. In benzamidine 47: anilinecarbonitrile fragment is blue, aromatic amine attaching to triple bond of carbonitrile, is pink.

The authors of publication [29] examined the reaction of **TCNE** with benzamidine **42** (Scheme 10) in an ethyl acetate medium at room temperature for 5 h. This interaction resulted in the formation of three compounds: dihydroimidazole derivative **55** with a yield of 12%, quinazoline **50** with a 37% yield, and iminodihydroquinazoline **52** with 2% yield. The chemical process was presumed to proceed through the tautomeric form **47′** in two pathways. The first involves the attachment of the primary amine to the **TCNE** in a Michael addition. Subsequently, both prussic acid (compound **49**) and malononitrile (compound **51**) can be eliminated. Subsequent intramolecular cyclization of the intermediates **49**, **51** yield the final products **50**, **52** through imine and secondary amine pathways, respectively. In the second pathway, tricyanovinylation of the secondary amine **47′** through the intermediate **53** is followed by intramolecular cyclization **54** according to the Thorpe–Ziegler type, resulting in the formation of the final product **55** (Scheme 10).

**Scheme 11:**
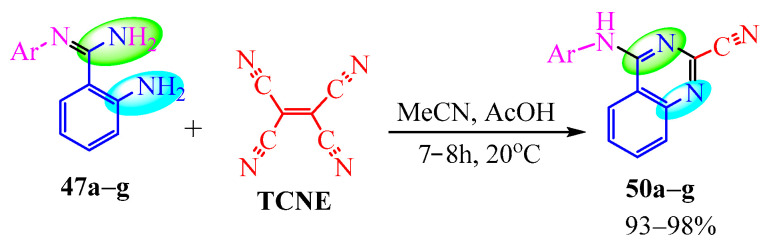
Conditions aimed at increasing the yield of the product **50**. Colored circles mark the reaction centres in molecules **47a**–**g**.

The authors of [29] achieved an increase in the yield of arylaminoquinazoline **50** from 37% to 93–98% when conducting the same reaction in acetonitrile with the addition of 1 equivalent of acetic acid at room temperature for 7–8 h (Scheme 11).

The results of this synthesis under the conditions described above (Scheme 11) are shown in Table 3.

**Scheme 12:**
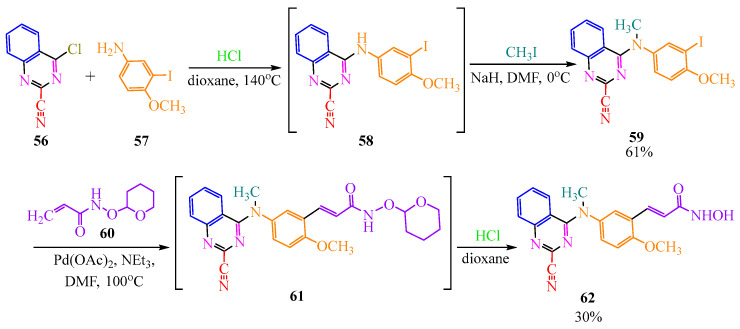
Additional method to obtain quinazoline derivative.

The authors of publication [30] obtained arylaminoquinazoline through an alternative pathway. In the first stage, they conducted the reaction of quinazoline **56** with aniline derivative **57** in an acidic medium in dioxane at 140 °C, followed by the methylation of the disubstituted amino group of intermediate **58** in dimethylformamide at 0 °C in the presence of methyl iodide and sodium hydride as a strong base for the elimination of hydrogen iodide. Target product **59** resulted in a yield of 61% (Scheme 12). In the second stage, they conducted a cross-coupling of this compound **59** with oxyacrylamide **60**. The reaction, catalyzed by a Pd(II) complex, was performed in the polar solvent dimethylformamide at 100 °C in the presence of triethylamine, which is essential for the elimination of the acrylamide vinyl hydrogen from **60**. The hydroxyamide group of the cross-coupling product **61** was then hydrolyzed in the presence of hydrochloric acid in dioxane, resulting in the final hydroxyamide **62** with a yield of 30% (Scheme 12).

The authors of article [30] evaluated the *N*-methyl derivative of heterocycles **61** and **62** on the HCT116 cell line (refer to the results in Table 12, “Biological activity of arylaminoquinazoline derivatives”, in Section 6, “Molecular Research”).

In addition, a significant number of arylaminoquinazolines demonstrate inhibitory activity against EGFR tyrosine kinase [31,32,33,34,35,36], Hoechst 33,342 [37], PARP [38], HCA [39], NAPE-PLD [40], nsP1 [41], PARP-1 [42], PDE-7 [43], and HFDPS [44]. Moreover, several derivatives also exhibit antibacterial activity [45,46].

**Scheme 13:**
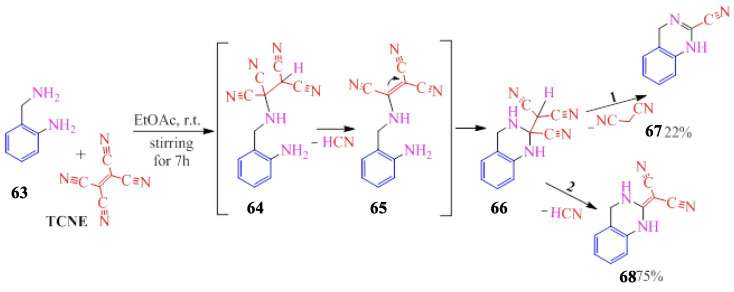
Interaction of 2-aminobenzylamine with **TCNE**.

The tricyanovinylation of 2-aminobenzylamine (Scheme 13) is carried out through the benzyl nitrogen [16], followed by the addition of an aromatic amino group via a Michael double bond. Compound **66** undergoes further chemical transformations along two pathways: one involving the elimination of malononitrile (compound **67**), and the other involving the production of prussic acid (compound **68**) (Scheme 13).

Additionally, **TCNE**-based syntheses enable the one-step formation of five-membered heterocycles, including pyrazoles [47,48], triazoles [49], and thiazoles [50].

**Scheme 14:**
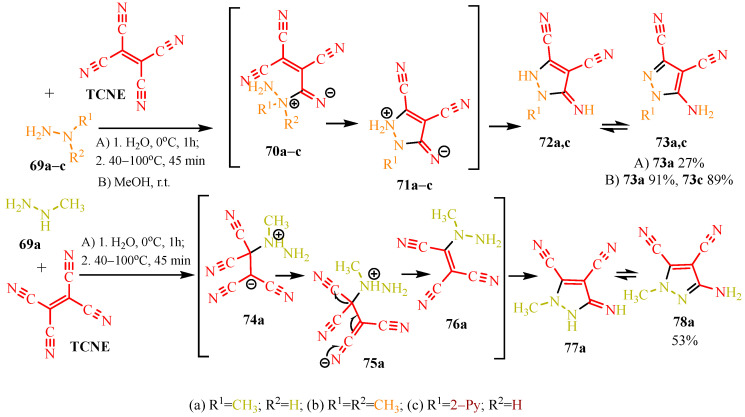
*N*-substituted hydrazines with **TCNE**.

Syntheses involving **TCNE** and hydrazine derivatives yield *N*-substituted pyrazoles [47,48]. According to publications [47,48], the cyclization of **TCNE** with hydrazines proceeds via tricyanovinyl intermediates. The authors of [47] conducted the synthesis using *N*-methylhydrazine **69a** in water, initially stirring for one hour at 0 °C followed by refluxing for 45 min in the temperature range 40–100 °C (see conditions A, Scheme 14). They propose a bifurcated reaction pathway involving the triple bond of the carbonitrile group and the double bond of **TCNE**. Addition of the *N*-methyl fragment **69a** to the carbonitrile of **TCNE** forms tricyanovinylimine **70a**, which cyclizes to iminopyrazole **71a** with elimination of cyanoacetic acid. Zwitterion **71a** converts to iminopyrazole **72a**, which tautomerizes to aminopyrazole **73a**, achieving a yield of 27%. Alternatively, attachment via the double bond of **TCNE** leads the authors of [47] to suggest the formation of tetracarbonitrile zwitterion **74a**, which tautomerizes to ketenimine **75a**, followed by elimination of cyanoacetic acid yielding tricyanovinylhydrazine **76a**. Intramolecular Thorp–Ziegler cyclization produces 3-iminopyrazole **77a**, which tautomerizes to 3-aminopyrazole **78a** with a yield of 53%, surpassing the yield of 5-aminopyrazole **73a** at 27%.

Our synthesis [48] involved **TCNE** and substituted hydrazines—dimethylhydrazine **69b** and pyridyl hydrazine **69c**—at room temperature in methanol for one day (see conditions B, Scheme 14). In contrast to the synthesis described above [47], the reaction with dimethylhydrazine **69b** [48] proceeds chemoselectively, yielding exclusively 5-aminopyrazole **73a** with a yield of 91%. Similarly, under these conditions [48], pyridyl hydrazine **69c** also yields pyrazole derivative **73c** with a yield of 89%. We hypothesized the formation of pyrazoles based on dimethylhydrazine **73a** and pyridyl hydrazine **73c** following the same pathway as described in publication [47], involving an addition of the substituted fragment of the corresponding hydrazine to the carbonitrile group of **TCNE**. Chromatographic methods and qualitative reactions with Prussian blue [48] confirmed that in the case of dimethylhydrazine **69b**, during the cyclization of tricyanovinylimine **70b** to iminopyrazole **72b**, acetonitrile is eliminated, while in the case of pyridyl hydrazine **69c**, cyanoacetic acid is eliminated (Scheme 14).

The conditions of syntheses for the original hydrazines **69a**–**c**, pyrazoles **73a**, **c**, and **78a** as well as their yields are presented in Table 4.

**Scheme 15:**
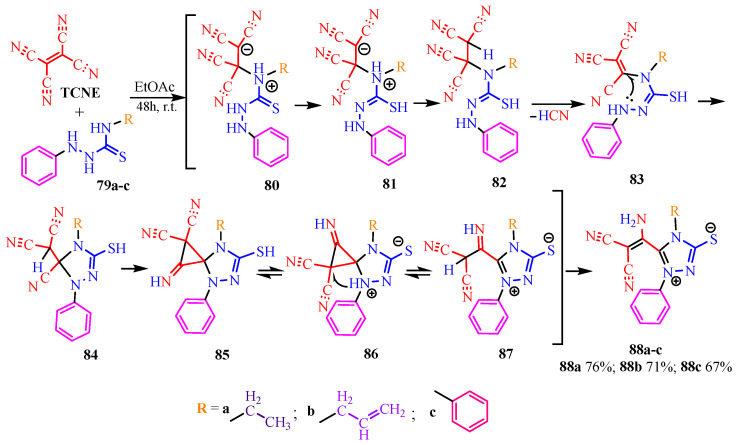
Mesoionic 1,2,4-triazolium-3-thiolate derivatives **88a**–**c**.

The reaction of **TCNE** with substituted phenylthiosemicarbazones (**79a**–**c**) [49] is quite unusual (Scheme 15). It is proposed that thiocarbazones **79a**–**c**, through zwitterion **80** and its tautomeric form **81**, protonate **TCNE** to form the tetracyanoethyl derivative **82**. This derivative undergoes elimination of hydrogen cyanide, yielding a tricyanovinyl intermediate **83**, which then undergoes intramolecular cyclization to form triazole **84**. Subsequently, a Thorpe–Ziegler-type spiro attachment of the malononitrile fragment to the cyano group in molecule **84** produces dicyanocyclopropanimine **85**. A 1,3-hydride transfer occurs, leading to the formation of an unstable zwitterion **86**, which opens the cyclopropane during the nucleophilic attack by the malononitrile fragment on the three-membered ring. The resulting iminomalononitrile **87** then tautomerizes to aminomalononitrile **88a**–**c**. The reaction was conducted in ethyl acetate at room temperature for 48 h. Based on the yields presented in Scheme 15, it can be inferred that the formation of the target 1,2,4-triazolium-3-thiolates decreases with increasing multiplicity and aromaticity of the substituents on the thiosemicarbazones.

**Scheme 16:**
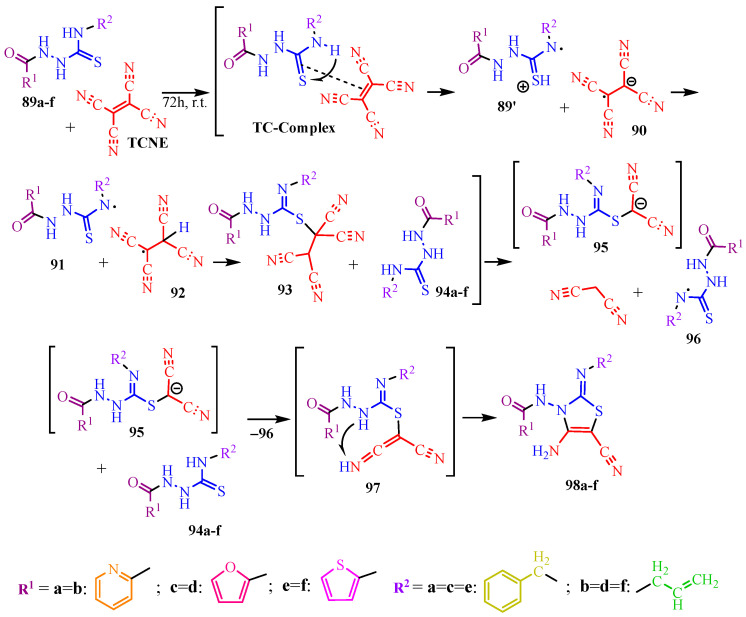
Formation of tetra-substituted thiazoles **98a**–**f**.

The reaction of TCNE with disubstituted thiosemicarbazides proceeds differently [50]. The authors of [50] propose that the initial stage involves a complexation reaction (TC–complex). This is followed by the decomposition of the complex into the thiosemicarbazide cation radical (**89′**) and tetracyanoethane (**90**). The cation radical **89′** then protonates the anion radical **90**, resulting in the formation of two radical species (**91** and **92**). The addition of the tetracyanoethane radical (**92**) to the multiple bond of radical **91**, accompanied by the redistribution of electron density, yields the tetracyanoethyl derivative **93**. This derivative is subsequently protonated by a second thiosemicarbazide molecule (**94a**–**f**), leading to the elimination of malononitrile and the formation of anion **95**. The anion **95** is again protonated by molecule **94a**–**f** to produce ketenimine **97**. The subsequent intramolecular cyclization—via the addition of an amino group to the multiple bond of ketenimine—results in the formation of the target thiazoles (**98a**–**f**) (Scheme 16).

The reaction of **TCNE** with thiosemicarbazides (**89a**–**f**) was conducted in five different solvents: tetrahydrofuran, dichloromethane, benzene, acetonitrile, and dioxane. The reactions in acetonitrile and dioxane have been previously studied [50], and a notably favorable result was observed in tetrahydrofuran. The results are summarized in Table 5.

**Scheme 17:**
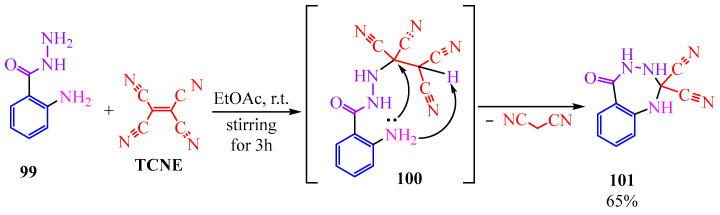
Interaction of **TCNE** with anthranilic acid hydrazide.

The reaction of **TCNE** with anthranilic (*o*-aminobenzoic) acid hydrazide **99** offers the opportunity to access a seven-membered cycle that is challenging to obtain [51], holding interest in the realms of organic and pharmaceutical chemistry as a potential antibacterial, antiviral, and psychotropic agent [52,53,54]. The interaction (Scheme 17) takes place through the terminal nitrogen of the hydrazide. Subsequent to the Michael addition (**100**), the elimination of malononitrile results in the formation of dicyanoazepine **101** (Scheme 17).

## 4. Heterocyclic Derivatives via Tricyanovinyl Intermediates

**Scheme 18:**
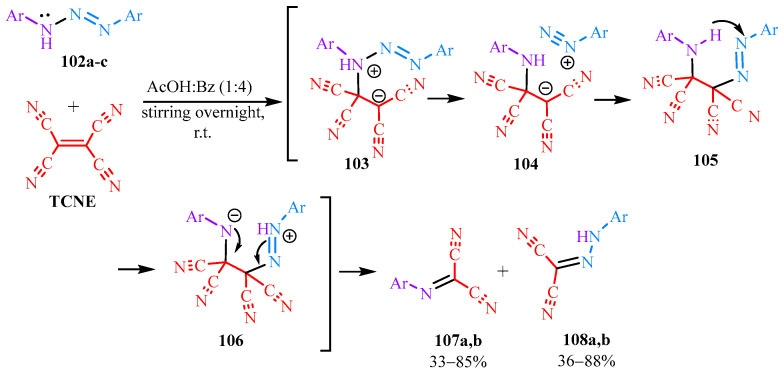
Interaction of **TCNE** with 1,3-aryltriazenes.

The interaction of **TCNE** with triazenes **102a**–**c** [55] is presumed to proceed through the rearrangement of intermediates **103**–**106**, followed by cleavage of bond **81** resulting in the formation of malonitriles **107a**,**b** and **108a**,**b** (Scheme 18).

The syntheses based on **TCNE** and triazenes **102a**–**c** have demonstrated that symmetric aryltriazenes **102a**,**b** yield Schiff’s bases **107a**,**b** and hydrazones **108a**,**b** with high yields (85–89%), whereas the same compounds derived from asymmetric aryltriazenes **102c** result in relatively lower yields in the range of 33–58%. The original compounds and the products are presented in Table 6.

Structures **108a**,**b** were found to affect the oxidative phosphorylation in mitochondria from rat liver, Paracoccus denitrificans bacteria, Candida albicans, yeast, and animal leukemia cells P388 [56].

**Figure 2:**
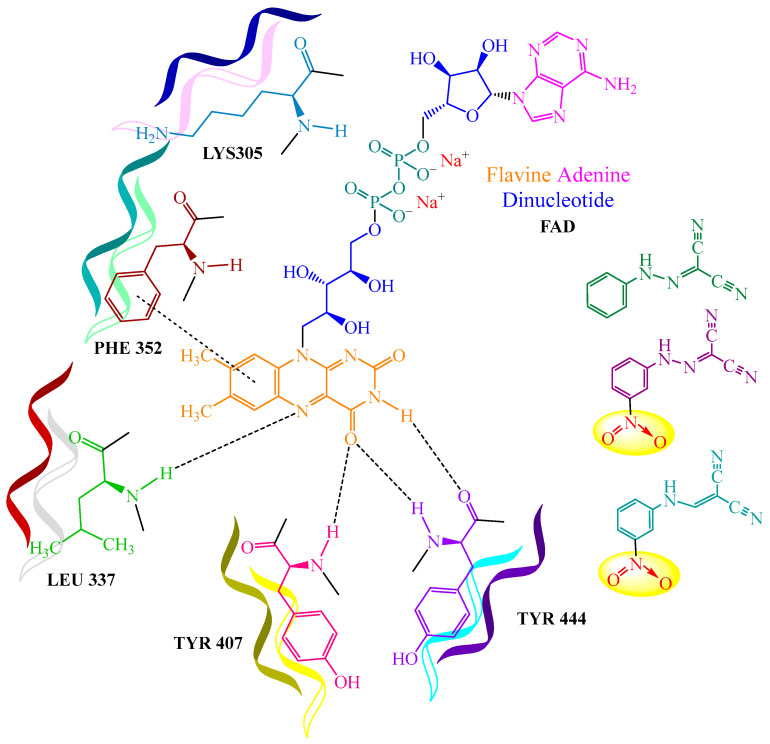
Binding of dicarbonitriles to the FAD center of monoamine oxidase. Colorful ribbons indicate the protein fragments of monoamineoxidase (MAO); nitro-groups are highlighted in yellow circle.

Additionally, compounds **108a**,**b** exhibited a notably significant MAO inhibitory activity (against monoamine oxidase, which promotes the catabolism of monoamines and catalyzes the synthesis of neurotransmitters and hormones in the body) [57], (refer to IC_50_ results in Table 13, “MAO inhibitory activity of the compounds”, in Section 6, “Molecular Research”). The authors of publication [57] also constructed a graphical model illustrating the binding sites of dicarbonitriles with the FAD center of monoamine oxidase (see Figure 2, “Binding of dicarbonitriles to the FAD center of monoamine oxidase”, below).

**Scheme 19:**
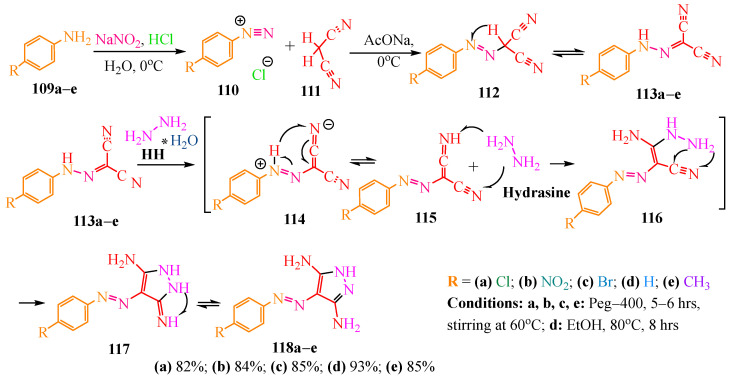
Synthesis of pyrazole based on hydrazine hydrate. * indicates binding between water and hydrazine in hydrazine hydrate.

In publication [58], derivatives of carbonohydrazonoyl dicarbonyl **113** were synthesized from the diazo compound following Scheme 19. Initially, diazonium salt **110** was prepared using standard diazotization conditions, where an aqueous solution of sodium nitrite was slowly added to an aqueous solution of arylamine **109** in hydrochloric acid at 0 °C to prevent decomposition. Subsequently, malononitrile **111** in sodium acetate was added to diazonium chloride **110** at 0 °C to minimize rapid nitrogen release. The resulting azo compounds **112** then underwent tautomerization to yield the final products **113a**–**e**.

In article [59], the authors employed carbonohydrazonyl dicarbonitriles **113a**–**e** in the synthesis of pyrazole derivatives **118a**–**e** using hydrazine hydrate (**HH**) in PEG-400 (polyethylene glycol 400). Conversely, in article [60], the same synthesis was conducted in EtOH according to Scheme 19. It is postulated that the basicity of **HH** facilitated the transformation of structure **113** into ketenimine **114**. Subsequent addition of hydrazine to tautomeric form **115** produced enamine **116**, and its intramolecular cyclization led to iminopyrazolone **117**. Tautomerization of the latter resulted in the formation of the target pyrazole **118** (Scheme 19).

However, the obtained pyrazoles **118a**–**e** did not exhibit significant MAO inhibitory activity compared to the initial reagents **113a**–**e** used in their synthesis.

**Scheme 20:**
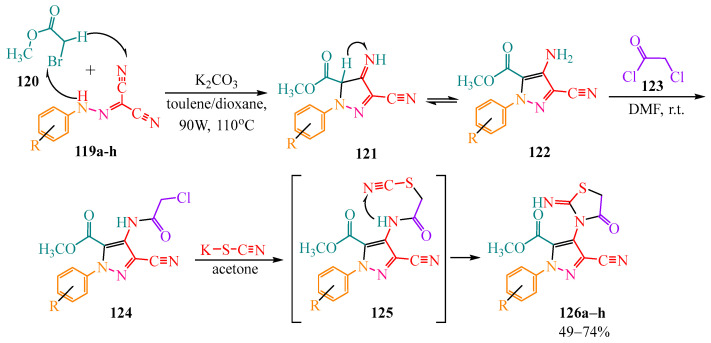
Synthesis of pyrazole based on -bromomethyl acetate. In intermediate **121**: arrow indicates tautomerization; In intermediate **125**: arrow indicates intermolecular cyclization.

The cyclization of dicarbonitriles **119a**–**h** into pyrazoles is also described in publications [61,62]. The synthesis was carried out using bromomethyl acetate **120** in the presence of potassium carbonate—which is essential for the elimination of hydrogen bromide—in a toluene/dioxane system [61] or in toluene [62] with microwave irradiation at 90 W and 110 °C (Scheme 20). In article [61], the authors performed further modifications with heterocycle **122**. This was accomplished by adding chloroacetic acid chlorohydride **123** to heterocycle **124** in a sufficiently basic solvent dimethylformamide (DMF), which is necessary for hydrogen elimination from amine **122** to form amide **124**. The reaction was conducted at room temperature to prevent the rapid release of hydrogen chloride. Potassium thiocyanate was added to the monosubstituted amide **124** in an acetone solution, leading to the formation of thiocyanoacetamide **125**, which cyclized into oxothiazolidines **126a**–**h** (Scheme 20).

The yields of the obtained pyrazoles are shown in Table 7.

**Scheme 21:**
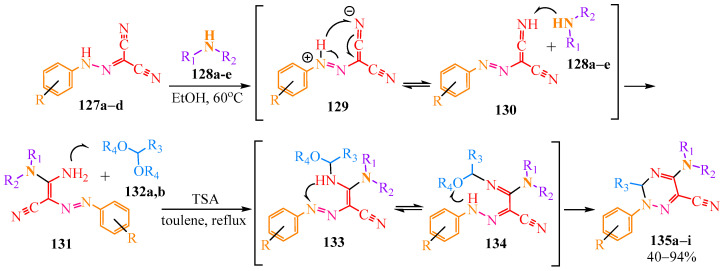
Synthesis of triazines **135a**–**i**.

Dicarbonitriles **102a**–**d** were also utilized in the two-stage synthesis of triazines with yields ranging from 40 to 94% [62] (Scheme 21). In the first stage, the authors of article [62] conducted the reaction with secondary amines **128a**–**e** in ethanol at 60 °C. It is presumed that the tautomeric form of dicarbonitriles **129**, stabilized by the basic amines **128a**–**e** (analogous to Scheme 19), interacted with them to form enamine **131**. Acetals **132a**,**b** were added to this compound **131** when heated in toluene in the presence of p-toluenesulfonic acid (TSA) as acetals are hydrolyzed in an acidic medium. This process resulted in the formation of amino-acetal **133**, which tautomerized to imino-acetal **134**. The intramolecular cyclization of **134** led to the formation of triazines **135a**–**i** (Scheme 21).

Original compounds: dicyanopyrazoles **127a**–**d**, disubstituted amines **128a**–**e**, target triazines **135a**–**i**, and their yields are presented in Table 8.

**Scheme 22:**
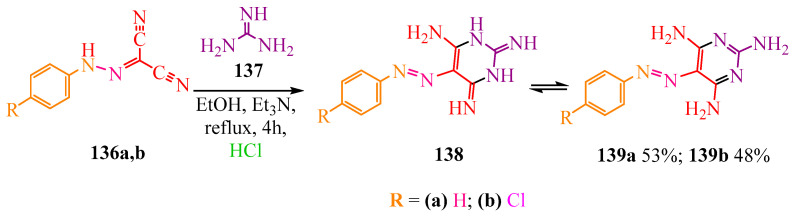
Synthesis of pyrimidines **139a**,**b**.

The authors of publication [63] obtained pyrimidine derivatives **139a**,**b**, which were evaluated for antitumor activity against four cell lines. Doxorubicin was used as a positive control (see Table 14, “Antitumor activity of compounds” in Section 6, “Molecular Research”). Heterocycles **139a**,**b** were synthesized from dicarbonitriles **136a**,**b** and carbamide **137**. The latter, **137**, reacted with hydrazone dicarbonitriles **136a**,**b**, resulting in the formation of imine **138**, which subsequently tautomerized to yield the target compounds **139a**,**b** (Scheme 22).

The resulting compound is also the strongest inhibitor of folic acid [64], and its derivatives are inhibitors of dihydrofolate reductase [65,66].

**Scheme 23:**
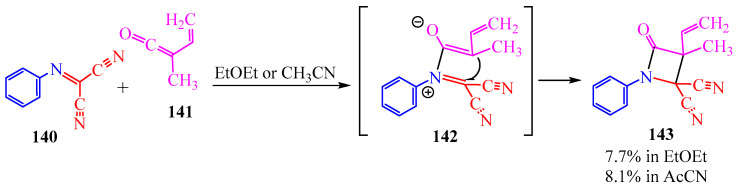
Synthesis of γ-lactam.

Dicarbonitrile **140** (Scheme 23) is no less interesting to create γ-lactam **143**. In publication [67], this heterocycle was derived from vinyl methylketene **141**. In diethyl ether the yield was 7.7%, in acetonitrile, 8.1% (Scheme 23).

**Scheme 24:**
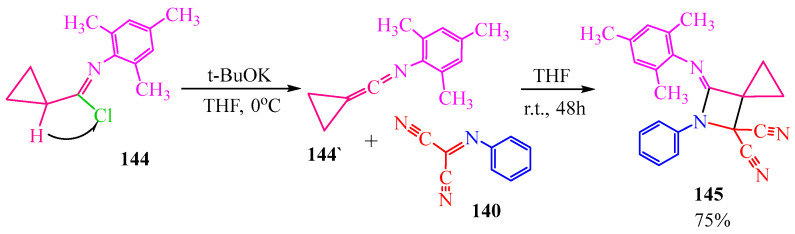
Synthesis of azaspiro [2,3]-hexane.

The synthesis of the azetidine derivative via compound **140** is also described in publication [68]. The authors of [68] initially obtained ketenimine **144′** from *N*-mesitylcyclopropancarbimidoyl chloride **144** in the presence of the strong base potassium tert-butoxide to eliminate hydrogen chloride. This reaction occurred in tetrahydrofuran at 0 °C to prevent the decomposition of cyclopropane. Cyclopropylidene **144′** then underwent a [2+2]-cycloaddition reaction with carbonimidoyl dicyanide **140**, resulting in the formation of azaspirohexane **145** (Scheme 24).

## 5. Synthesis of Optically Active Compounds via Pericyclic Reactions with TCNE

**Scheme 25:**
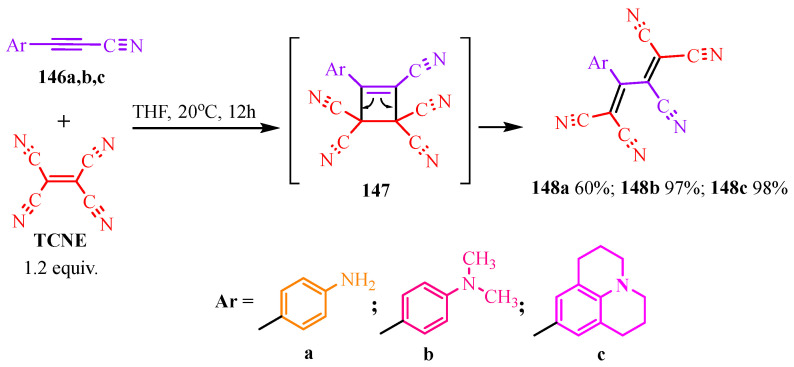
The [2+2] cycloaddition.

**TCNE**-based syntheses of optically active butadiene 1,1,4,4-tetracarbonitriles are utilized to create chromophores [69,70,71] and photosensitizers [72,73] that have widespread applications in organic chemistry [70,71,72,73]. According to publication [73], disubstituted acetylenes **146a**–**c** undergo [2+2]-cycloaddition with **TCNE**, leading to the formation of cyclobutenes **147** (Scheme 25). In this case, the donor, which is a substituent of the aromatic ring, contributes to the displacement of electron density, resulting in the cleavage of the four-membered cycle **147** into derivatives of butadiene **148a**–**c** (Scheme 25).

The synthesized compounds absorb UV radiation within the wavelength range of 300–550 nm. Among them, the hexahydrobenzoquinoline derivative **148c** exhibits the most significant bathochromic shift (up to 500 nm) and the highest radiation intensity. Compound **148c** proves to be highly effective for use as a photosensitive material due to its strong fluorescent response and high yield (98%) under relatively simple synthesis conditions.

**Scheme 26:**
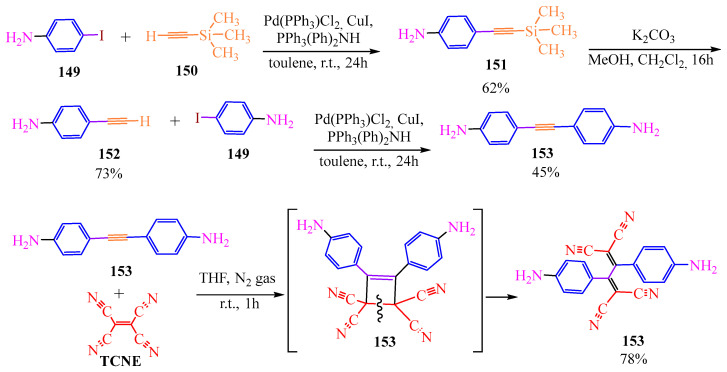
Synthesis of calorimetric sensors for sulfide ions.

Pericyclic reactions were also employed in the synthesis of calorimetric sensors for sulfide ions [72]. Initially, 4-iodoaminobenzene **149** underwent cross-coupling catalyzed by Pd(II) with trimethylsilyl acetylene **150** in the presence of a disubstituted amine and copper iodide as a buffer. The authors of [72] subsequently removed the trimethylsilyl group from compound **151** using potassium carbonate in a mixture of methanol and dichloromethane for 16 h. Thereafter, the monosubstituted acetylene derivative underwent a cross-coupling reaction with 4-iodoaminobenzene **153** under conditions similar to the preparation of anilyl acetyltrimethylsilane **151**. This reaction led to the formation of bis-para-anilyl-acetylene **153**. Reaction of compound **153** with **TCNE** via [2+2]-cycloaddition intermediate **154** resulted in the formation of butadiene-tetracarbonitrile **155** (Scheme 26).

## 6. Molecular Research

The authors of article [22] measured the light absorption of tricyanovinyl derivatives derived from aniline and 2-methylindole in six different solvents: hexane, toluene, dichloromethane (DCM), methanol (MeOH), dimethylformamide (DMF), and dimethyl sulfoxide (DMSO). The results indicate a significant correlation between solvent polarity and the observed bathochromic shift. The data are summarized in Table 9.

As an example of the effect of solvent polarity on the shift of light absorption to longer wavelengths, solutions of tricyanovinylated DMA in these solvents are presented in [22] (Figure 1).

The derivatives of reactions between TCNE and aromatic amines considered in this article have a wide range of biological activities and are quite convenient in analytical studies.

Thus, the ability of TCNE to complex with nitrogen- and oxygen-containing compounds was used by the authors of [28] to establish spectrometric characteristics that determine the behavior of drugs in the human body. Identification of TCNE complexes by spectroscopic methods allows us to determine the mechanism of the drug’s action, the binding site of the active ingredient molecule to the biological target, physical and thermodynamic properties, and to quantify the purity of the drug. Moreover, TCNE-based complex compounds are active against both Gram-positive and Gram-negative bacteria. The main advantage of the method proposed by the authors of article [28] is the possibility of conducting the study without isolating the active substance from the drug substance, which is quite time-consuming and accompanied by significant losses of the target product. In the experiment described in article [28], eight drugs were used, as outlined below:

1. **Glycoside**, which is used for the treatment of type 2 diabetes through blood glucose control [74];
2. **Papaverine hydrochloride**, which aims to treat renal colic as well as gastrointestinal, bile duct, and ureteral spasms [75];
3. **Pilocaprine hydrochloride**, which pharmacologically stimulates exocrine glands that promote sweating, salivation, lacrimation, and gastric and pancreatic secretion. Additionally, this drug has been used for a long time to treat glaucoma [76];
4. **Procaine hydrochloride**, which reduces pain from intramuscular injections of penicillin and is used in dentistry [77];
5. **Aminoantipyrine**, which finds application in pharmacological, biological, biochemical, and analytical studies, and can reduce bleeding and denature bovine hemoglobin [78];
6. **Sulfamethoxazole**, which is a cheap and effective synthetic antibiotic used against most Gram-positive and Gram-negative bacteria [79];
7. **Sulfathiazole**, which has the same characteristics as Sulfamethoxazole [80];
8. **Simvastathathione**, which aims to reduce cholesterol levels and the risks of atherosclerosis and myocardial infarction, additionally possessing anti-inflammatory effects on the skin and crack healing [81].

The authors of [28] also determined the thermodynamic parameters (Table 10) and found their correlations. The enthalpy value is strongly correlated with entropy and Gibbs energy. The correlation between entropy and Gibbs energy is especially pronounced.

To investigate the obtained complex compounds, the authors of [28] performed spectrophotometric measurements, stoichiometric titration, and determined the thermodynamic parameters of the studied compounds. During light absorption measurements, the authors of [28] noticed an interesting phenomenon: the peaks of TCNE complexes with ***Drugs 1***, ***4***, ***5***, and ***7*** have one absorption maximum, while the ***Drug 2***, ***3***, ***6***, and ***8*** complexes split into two peaks. The authors believe that one maximum of light absorption corresponds to the n→π* transition, further corresponding to the interaction of the TCNE double bond with the drug by one reaction center—the unshared electron pair of the N or O atom with (type A interaction, Table 11) two maximums—the π→π* transition, implying the interaction of the TCNE double bond by two reaction centers—the unshared electron pair of the N or O atom and the electron density of the aromatic ring or C=O (type B interaction, Table 11).

The authors of article [30] evaluated the derivatives of compound **56** for its cytotoxic activity against the HCT116 colon cancer cell line (Table 12). The HCT116 cells were cultured in RPMI-1640 medium, which lacks growth factors, necessitating supplementation with 10% fetal calf serum (FCS) and 1% glutamine. The FCS was pretreated with the antitumor agent mitomycin C and irradiated with ultraviolet light to inhibit cell division, thereby allowing it to serve only a metabolic function to support HCT116 cell growth. The colon cancer cells were incubated at 37 °C in a humidified atmosphere with 5% CO_2_, which likely helped neutralize ammonia generated from the decomposition of glutamine. After 24 h, the medium containing the cells was treated with the test compounds at various concentrations. The cells, along with the quinozaline derivatives, were incubated for an additional 72 h. Subsequently, 100 µL of CellTiter-Glo Reagent was added to each well to assess the biological activity of the compounds via luminescence and spectrophotometric analysis. The CellTiter-Glo Reagent produces a luminescent signal through its interaction with adenosine triphosphate (ATP) molecules, the intensity of which is directly proportional to the number of viable HCT116 cells. A reduction in luminescence indicates the inhibitory effect of the tested compounds on tumor cell proliferation.

The compounds tested demonstrated significant antitumor activity against the HCT116 cell line, with the results summarized in Table 12.

**TCNE** and triazene derivatives, specifically hydrazonemalononitriles, have demonstrated significant MAO inhibitory activity (monoamine oxidase inhibition), which plays a crucial role in the catabolism of monoamines and the regulation of neurotransmitter and hormone synthesis in the body [57]. MAO inhibitors are widely used in the treatment of neurodegenerative diseases such as Alzheimer’s and Parkinson’s, as well as in managing conditions like anxiety, panic attacks, social phobia, and post-traumatic stress disorder.

The MAO inhibitory activity of hydrazonemalononitriles was assessed through fluorometric detection of hydrogen peroxide (H_2_O_2_), a by-product generated during the oxidative deamination of amines by monoamine oxidase (MAO). In this assay, horseradish peroxidase (acting as a model enzyme), tyramine (the substrate to be deaminated), resazurin (a fluorescent indicator for H_2_O_2_), and the test compound were used. Resazurin and tyramine, both dissolved in phosphate buffer solution, along with the test compound dissolved in DMSO, were sequentially added to a solution of horseradish peroxidase in phosphate buffer. The mixture was incubated in a microplate well at 37 °C for 30 min, after which fluorometric measurements were performed.

Upon interaction between horseradish peroxidase and tyramine, H_2_O_2_ is produced [82], which subsequently oxidizes resazurin to resorufin. This reaction is accompanied by a color change from blue to fluorescent pink, with an excitation maximum at 530–570 nm and an emission maximum at 580–590 nm [83]. In the presence of an active test compound, the formation of H_2_O_2_ is inhibited, resulting in a cessation of the color change and a decrease in both excitation and emission intensity.

The results of these assays are summarized in Table 13. These data indicate that the nitrobenzene derivative exhibit the most potent inhibitory effect with the lowest measurement error.

The authors of publication [57] also developed a graphical model illustrating the binding sites of dicarbonitriles to the flavin adenine dinucleotide (FAD) center of monoamine oxidase (Figure 2). The study revealed that the nitro group within the dicarbonitrile structures plays a crucial role in binding to this coenzyme (FAD).

**TCNE**-based pyrimidines [63] were evaluated for their antitumor activity against four cell lines: MCF-7 (breast cancer), NCI-H460 (lung cancer), SF-268 (brain tumor), and WI-38 (normal pulmonary fibroblasts) (Table 14). These cell cultures were maintained in RPMI-1640 medium, supplemented with glutamine, heat-inactivated fetal bovine serum (FBS)—which supports cell viability and division—and antibiotics (penicillin and streptomycin) to prevent contamination from FBS. The cultures were incubated for 24 h at 37 °C in a humidified atmosphere with CO_2_ to neutralize the ammonia (NH_3_) produced during glutamine degradation.

Following incubation, the cells were stained with sulforhodamine B, a dye that binds to cellular proteins and forms a red fluorescent complex upon laser excitation, enabling the quantification of cell numbers [84]. Each test compound was added to the cultured and stained cells at five different concentrations, with a maximum concentration of 150 μM. After 48 h, the cells treated with the test compounds were fixed, washed with 0.5% DMSO, and stained. The stained cells were then dissolved in DMSO for subsequent measurement of light absorption at 492 nm (noting that sulforhodamine B exhibits maximum absorption at 565 nm and maximum fluorescence emission at 568 nm [84]).

A reduction in light absorption intensity indicated a decrease in cancer cell viability. Doxorubicin, tested under similar conditions, served as a reference compound. **TCNE**-derived pyrimidines exhibited moderate antitumor activity against the tested cell lines. The results are summarized in Table 14.

## 7. Conclusions

The products resulting from the reaction of tetracyanoethylene (TCNE) with arylamines have attracted significant attention across diverse scientific disciplines, including optics, pharmacology, and organic chemistry. Moreover, this reaction follows alternative pathways in several notable cases:

- TCNE undergoes a [3+2]-cycloaddition with subsequent rearrangement and C-C bond cleavage when reacting with triazenes;
- Addition of TCNE to 2-amino-N-benzamidine occurs through multiple reaction centers, followed by intramolecular cyclization. One instance suggests the elimination of both hydrogen cyanide and malononitrile;
- Tricyanovinylation selectively proceeds with primary arylamines such as 2-aminobenzylamine and anthranilic acid hydrazide. In these cases, TCNE adds to benzylamine and to the terminal nitrogen of the hydrazide, respectively;
- Aromatic amino groups in compounds containing alkynes, butadiene moieties, and nitroso groups do not participate in the reaction with TCNE uniformly. For instance, disubstituted alkyne derivatives undergo [2+2]-cycloaddition followed by cyclobutene cleavage, whereas compounds with butadiene moieties undergo [2+4]-cycloaddition to form stable six-membered cycles. Certain arylamine molecules containing both amino and nitroso groups add to TCNE, leading to N-N bond cleavage and formation of malononitrile derivatives.

The deviations from the standard tricyanovinylation scheme in these reactions warrant further investigation into the chemistry of TCNE with arylamines. TCNE-based syntheses show promising applications for the future, including the non-destructive analysis of drugs in pharmaceutical laboratories. Additionally, the facile synthesis of electrically conductive butadienetetracarbonitriles with high luminescent efficiency (97–98% yield via straightforward mixing in tetrahydrofuran at room temperature) holds potential for the development of photoelectronic and photosensitive materials. TCNE’s reactivity also facilitates the production of complex biologically active heterocyclic structures with high yields (95–98%) using a relatively simple technique (synthesis of arylaminoquinazolines by mixing TCNE with benzamidine in acetonitrile at room temperature), making it suitable for adoption in various pharmaceutical applications.

## Figures and Tables

**Table 1:** Results of tricyanovinylation reaction in three different conditions.

Original Compounds	Products	Lipase	DES	Protease
		Yield %, (min)	Yield %, (min)	Yield %, (min)
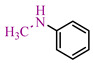 **33a**	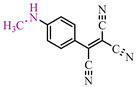 **34a**	80, (5)	89, (5)	79, (5)
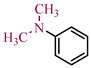 **33b**	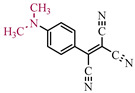 **34b**	84, (5)	89, (5)	74, (5)
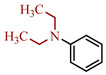 **33c**	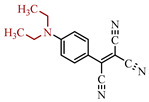 **34c**	85, (5)	87, (5)	84, (5)
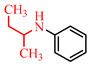 **33d**	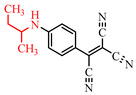 **34d**	70, (10)	74, (10)	68, (10)
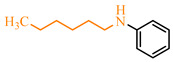 **33e**	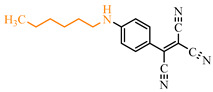 **34e**	75, (7)	84, (5)	74, (7)
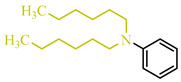 **33f**	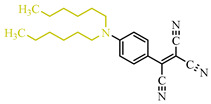 **34f**	78, (5)	87, (5)	75, (5)
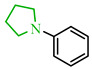 **33g**	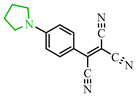 **34g**	83, (10)	80, (10)	79, (10)
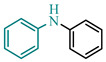 **33h**	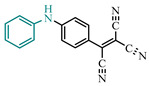 **34h**	75, (12)	82, (12)	73, (15)
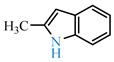 **35i**	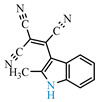 **36i**	75, (25)	75, (15)	72, (20)
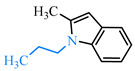 **35j**	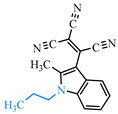 **36j**	78, (20)	87, (15)	80, (15)
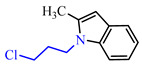 **35k**	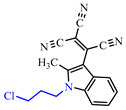 **36k**	76, (15)	81, (15)	75, (15)
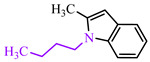 **35l**	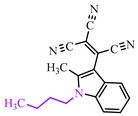 **36l**	80, (15)	85, (10)	80, (15)

**Table 2:** Optimization of catalysts.

Catalyst	Time (min)	Yield (%)
ChCl–malonic acid	25	70
ChCl–oxalic acid	15	70
ChCl–urea	5	89
Glycerol	30	80
Urea	60	20
ChCl	50	50
ChCl–ethanol	60	40
ChCl–H_2_O	60	75
ChCl–DCM	60	80
ChCl–urea–ethanol	60	10
ChCl–urea–DCM	10	85
5% Protease	5	60
10% Protease	5	68
15% Protease	5	79
20% Protease	5	76
5% Lipase	5	66
10% Lipase	5	75
15% Lipase	5	80
20% Lipase	5	80

**Table 3:** Yields of arylaminoquinosalines **50a**–**g**.

Reagents	Target Products	Yields (%)
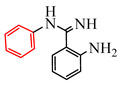 **47a**	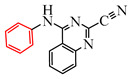 **50a**	97
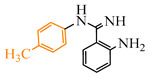 **47b**	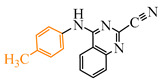 **50b**	98
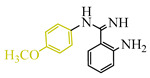 **47c**	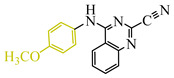 **50c**	97
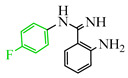 **47d**	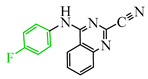 **50d**	98
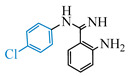 **47e**	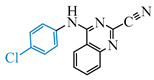 **50e**	95
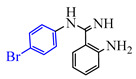 **47f**	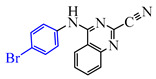 **50f**	97
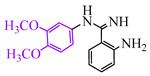 **47g**	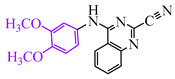 **50g**	93

**Table 4:** Conditions of syntheses and yields of *N*-substituted pyrazoles.

	Conditions	Original Hydrazine	Product and Yield (%)	
A	1. H_2_O, 0 °C, stirring 1 h; 2. Reflux, 40–100 °C 45 min	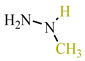 **69a**	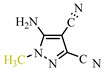 **73a**, 27%	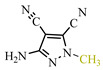 **78a**, 53%
B	MeOH, r.t.	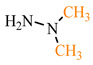 **69b**	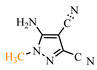 **73a**, 91%	
		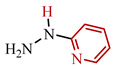 **69c**	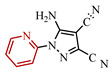 **73c**, 89%	

**Table 5:** Yields of synthetic products **98a**–**f** in different solvents.

Structure	Yield 88a–f, %				
	THF	CH_2_Cl_2_	Benzene	CH_3_CN	1,4-Dioxane
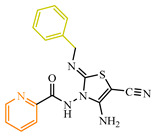 **98a**	79	36	33	40	56
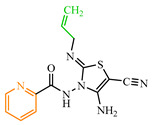 **98b**	71	29	30	38	53
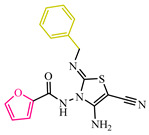 **98c**	74	34	31	39	54
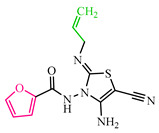 **98d**	68	28	27	37	51
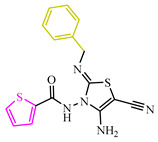 **98e**	77	34	31	39	54
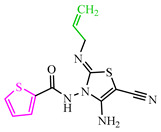 **98f**	70	28	27	36	51

**Table 6:** Yields of Schiff’s bases and hydrazones.

Reagents 77a–c	Schiff’s Bases 82a,b	Yields (%)	Hydrazones 83a,b	Yields (%)
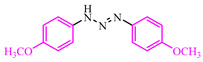 **102a**	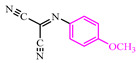 **107a**	85	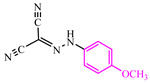 **108a**	88
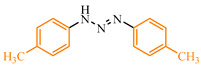 **102b**	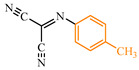 **107b**	89	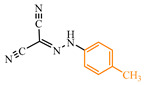 **108b**	89
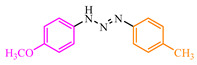 **102c**	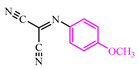 **107a**	33	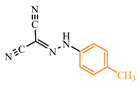 **108b**	36
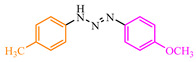 **102c**	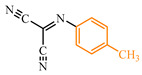 **107b**	58	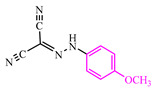 **108a**	58

**Table 7:** Pyrazole **126a**–**h** yields.

Reagents 119a–h	Target Products 126a–h	Yield, %
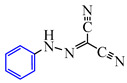 **119a**	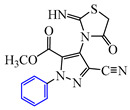 **126a**	55
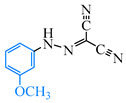 **119b**	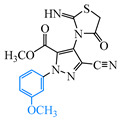 **126b**	70
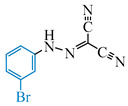 **119c**	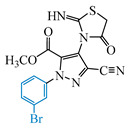 **126c**	67
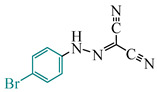 **119d**	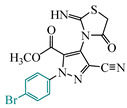 **126d**	49
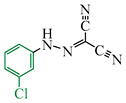 **119e**	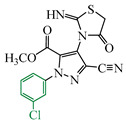 **126e**	72
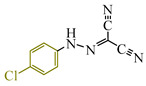 **119f**	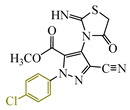 **126f**	49
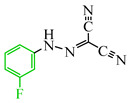 **119g**	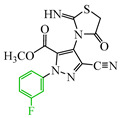 **126g**	49
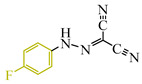 **119h**	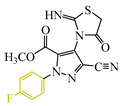 **126h**	74

**Table 8:** Reagents **127a**–**d**, **128a**–**e**, **132a**,**b**, target compounds **135a**–**i**, and their yields.

Reagents 127a–d	Reagents 128a–e	Reagents 132a,b	Target Products 135a–i	Yields (%)
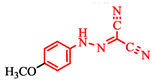 **127a**	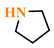 **128a**	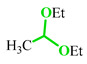 **132a**	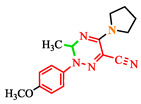 **135a**	40
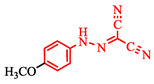 **127a**	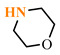 **128b**	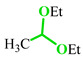 **132a**	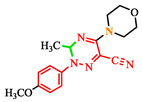 **135b**	70
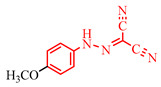 **127a**	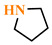 **128a**	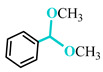 **132b**	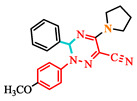 **135c**	81
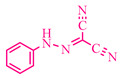 **127b**	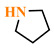 **128a**	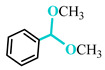 **132b**	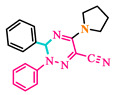 **135d**	81
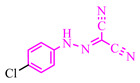 **127c**	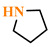 **128a**	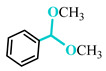 **132b**	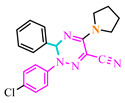 **135e**	67
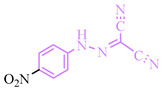 **127d**	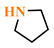 **128a**	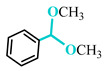 **132b**	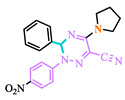 **135f**	73
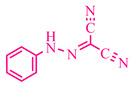 **127b**	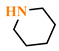 **128c**	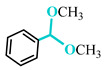 **132b**	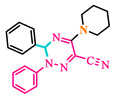 **135g**	80
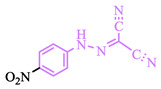 **127d**	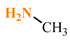 **128d**	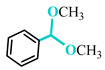 **132b**	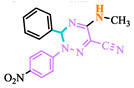 **135i**	52

**Table 9:** Absorption maxima for tricyanovinylated compounds.

Compound	Hexane (λ_abs_)	Toluene (λ_abs_)	DCM (λ_abs_)	MeOH (λ_abs_)	DMF (λ_abs_)	DMSO (λ_abs_)
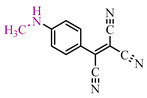	462	483	494	502	514	519
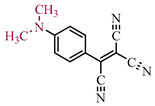	458	511	519	514	525	531
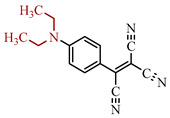	461	488	527	520	531	536
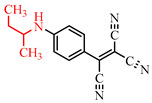	448	488	501	509	520	526
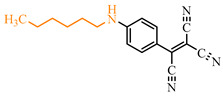	464	486	499	508	518	524
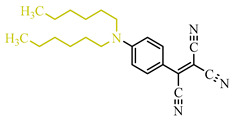	486	487	499	507	535	537
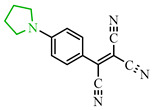	462	512	529	521	535	538
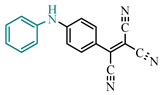	463	490	501	510	533	528
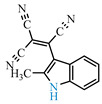	465	460	462	464	523	474
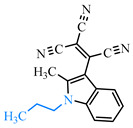	486	474	480	472	522	478
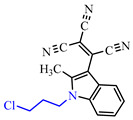	478	471	483	469	477	476
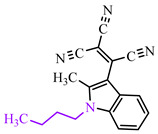	457	474	480	472	475	478

**Table 10:** Thermodynamic parameters of complex compounds.

Abbreviation	Drug, Weight (g/mol)	Complex	∆H* (JK^−1^ mol^−1^)	∆S* (JK^−1^ mol^−1^)	∆G* (JK^−1^ mol^−1^)
***Drug 1***	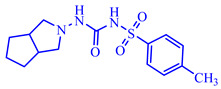 **Glicazide**, 323.41	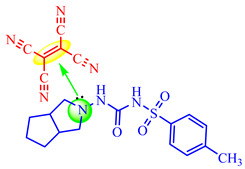	−20.483	−49.30	−57.92
***Drug 2***	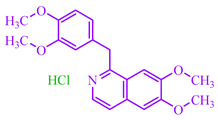 **Papaverine HCl**, 375.86	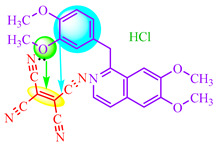	−8392	−67.26	11.715
***Drug 3***	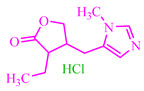 **Pilocaprine HCl**, 244.72	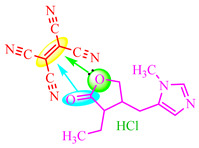	−69.344	113.07	−103.039
***Drug 4***	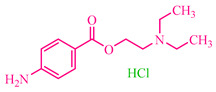 **Procaine HCl**, 272.78	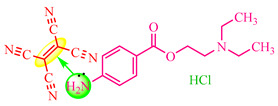	−93.675	196.38	152.196
***Drug 5***	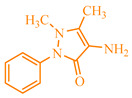 **4-Aminoantipyrine**, 203.24	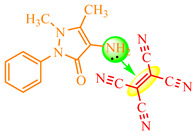	−2874	−106.75	28.938
***Drug 6***	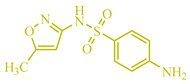 **Sulfamethoxazole**, 253.28	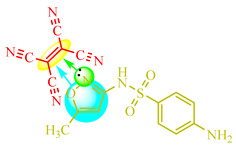	−2783	−92.19	24.690
***Drug 7***	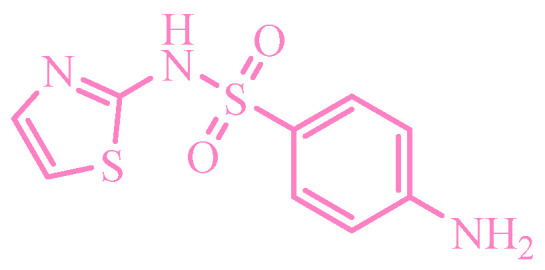 **Sulfathiazole**, 255.32	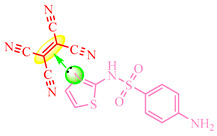	−2157	−108.66	30.224
***Drug 8***	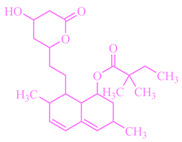 **Simvastation**, 255.32	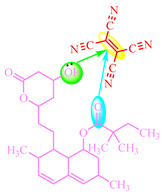	−4485	−90.96	−22.621

**Table 11:** (A) Non-splitting and (B) splitting absorption of bonds of synthesized complexes.

Type A Non-Splitting Interaction	Type B, Splitting Interaction
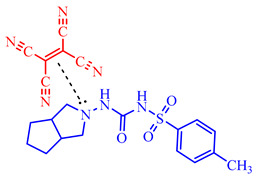 ***Drug 1***	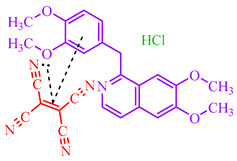 ***Drug 2***
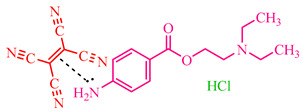 ***Drug 4***	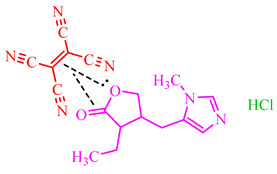 ***Drug 3***
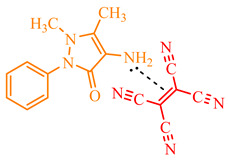 ***Drug 5***	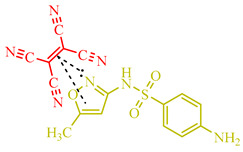 ***Drug 6***
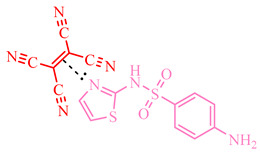 ***Drug 7***	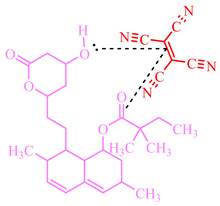 ***Drug 8***

**Table 12:** Biological activity of arylaminoquinazoline derivatives.

**Compound**	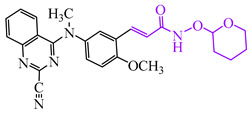	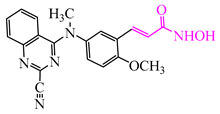
**GI_50_ HCT 16 (nm)**	1.5 ± 2.2	1.5 ± 0.07

**Table 13:** MAO inhibitory activity of the compounds.

**Compound**	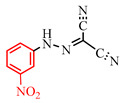	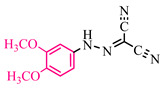	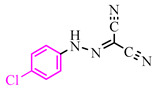	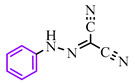	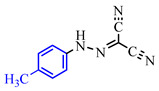
**hMAO IC_50_ mkM**	1.65 ± 0.17	10.7 ± 3.79	11.4 ± 6.79	9.57 ± 3.60	43.5 ± 18.5

**Table 14:** Antitumor activity of compounds.

Compounds	GI_50_ (mkmol/L)			
	MCF-7	NCI-H460	SF-268	WI-38
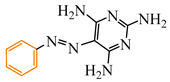	34.6 ± 5.2	33.1 ± 5.7	40.3 ± 7.5	65.5 ± 11.7
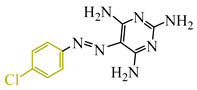	42.2 ± 8.2	44.4 ± 7.3	40.1 ± 7.3	na
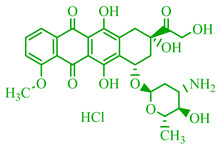 **Doxorubicin**	0.03 ± 0.008	0.07 ± 0.008	0.09 ± 0.007	>100

“na” indicates not active.

## Data Availability

Data on Spectra and High-Pressure Liquid Chromatography (HPLC) supporting reported results, can be found at “Supplementary Matherials”.

## Supplementary Materials

The following supporting information can be downloaded at: https://www.mdpi.com/article/10.3390/molecules29194727/s1, Supplementary File S1: Spectral data of the synthetysed compounds.
