# Supplementary material for: Reactions of Tetracyanoethylene with Aliphatic and Aromatic Amines and Hydrazines and Chemical Transformations of Tetracyanoethylene Derivatives
Source: Molecules. 2024 Oct 6;29(19):4727. doi: 10.3390/molecules29194727 (PMC11477996; doi:10.3390/molecules29194727)
Supplement: Supplementary file 1 [file molecules-29-04727-s001.zip › molecules-3225337-supplementary.pdf]

Supporting information

Reactions of Tetracyanoethylene with Aliphatic and Aromatic Amines and Hydrazines and Chemical Transformations of Tetracyanoethylene Derivatives

Elizaveta S. Ivanova,[1] Oleg E. Nasakin,[1] Margarita P. Osipova,[1] Yhtyyar Kadyrov,[1] Sergey V. Karpov,[1] Svetlana A. Markova,[1] Tatyana V. Vasilieva[1], Ekaterina I. Zazhivihina[1], Lubov A. Umanova[1], Yurii N. Mitrasov[2]*

[1]*Ulyanov Chuvash State University, Moskovsky pr., 15, Cheboksary 428015, Russia*

[2]*Organic and Pharmaceutical Chemistry Department, Yakovlev Chuvash State Pedagogical University, K. Marx Street, 38, 428000, Cheboksary, Russia*

**Corresponding author. Tel.: +7 903 345 57 33 (Oleg E. Nasakin); +7 961 340 01 81 (Elizaveta S.Ivanova).*

E-mail address: ecopan21@inbox.ru (O.E. Nasakin), lizachimic@mail.ru (E.S. Ivanova)

Spectral data of the synthetysed compounds

1. Tricyanoaniline derivatives 34b-g [22]

2-[4-(Dimethylamino)phenyl]-1,1,2-ethenetricarbonitrile (34b)

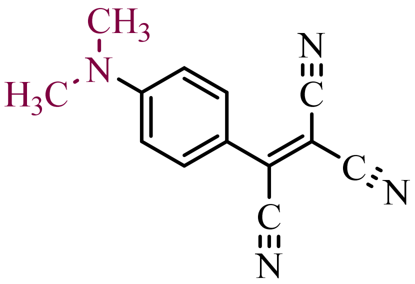

Purple solid; mp 174-176 °C (ethyl acetate); υ_{max}/cm^{-1} (KBr): 2211 (C≡N), 1608 (aromatic C=C) and 1384 (C-N); ^{1}H NMR (300 MHz, $CDCl_3$, Me_4Si) δ_H 8.06 (2H, d, J = 9.4 Hz, ArH), 6.74 (2H, d, J = 9.4 Hz, ArH), 3.22 (6H, s, 2xCH_3); MS = 223.5 (M+H).

2-[4-(Diethylamino)phenyl]-1,1,2-ethenetricarbonitrile (34c)

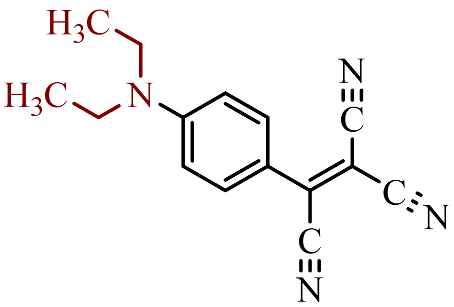

Purple solid; mp 164-166 °C (ethyl acetate); υ_{max}/cm^{-1} (KBr): 2211 (C≡N), 1604 (aromatic C=C) and 1384 (C-N); ^{1}H NMR (300 MHz, $CDCl_3$, Me_4Si) δ_H 8.06 (2H, d, J = 9.3 Hz, ArH), 6.73 (2H, d, J = 9.3 Hz, ArH), 3.54 (4H, q, J = 7 Hz, 2xCH_2), 1.28 (6H, t, J = 7 Hz, 2xCH_3); MS = 251.6 (M+H).

2-[4-(2-Butylamino)phenyl]-1,1,2-ethenetricarbonitrile (34d)

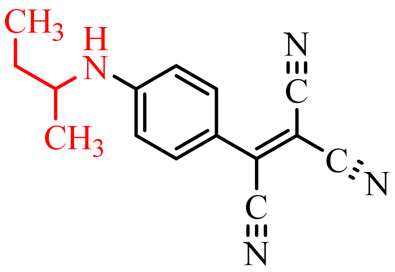

Purple solid; mp 139-141 °C (ethyl acetate); υ_{max}/cm^{-1} (KBr): 3327 (N-H), 2217 (C≡N), 1611 (aromatic C=C) and 1384 (C-N); ^{1}H NMR ($CDCl_3$, 300 MHz, Me_4Si) δ_H 8.03 (2H, d, J = 9.3 Hz, ArH), 6.63 (2H, d, J = 9.3 Hz, ArH), 4.96 (1H, bs, NH), 3.62 (1H, m, CH), 1.57-1.68 (2H, m, CH_2), 1.28 (2H, d, J = 6.6 Hz, CH_2), 0.99 (3H, t, J = 7.8 Hz, CH_3); ^{13}C NMR ($CDCl_3$, 125 MHz) δ_c 10.4, 20.1, 29.6, 50.4, 78.1, 113.4, 113.6, 113.9, 114.4, 118.1, 133.4, 137.9, 154.1; MS = 251.6 (M+H).

2-[4-(Hexylamino)phenyl]-1,1,2-ethenetricarbonitrile (34e)

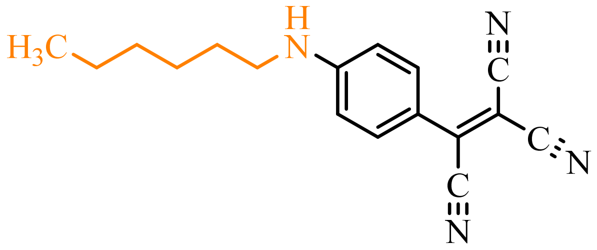

Purple solid; mp 100-102 °C (ethyl acetate); υ_{max}/cm^{-1} (KBr): 3342 (N-H), 2218 (C≡N), 1618 (aromatic C=C) and 1384 (C-N); ^{1}H NMR (300 MHz, $CDCl_3$, Me_4Si) δ_H 8.03 (2H, d, J = 9 Hz, ArH), 6.65 (2H, d, J = 9 Hz, ArH), 5.23 (1H, bs, NH), 3.30 (2H, t, J = 6.9 Hz, CH_2), 1.66 (2H, m, J = 6.9 Hz, CH_2), 1.33-1.41 (m, 6H), 0.91 (3H, t, J = 6.3 Hz, CH_3); ^{13}C NMR (125 MHz, $CDCl_3$) δ_c 14.0, 22.6, 26.6, 29.0, 31.4, 43.5, 78.0, 112.1, 113.7, 113.9, 114.5, 118.2, 133.4, 137.9, 154.9; MS = 279.7 (M+H).

2-[4-(Dihexylamino)phenyl]-1,1,2-ethenetricarbonitrile (34f)

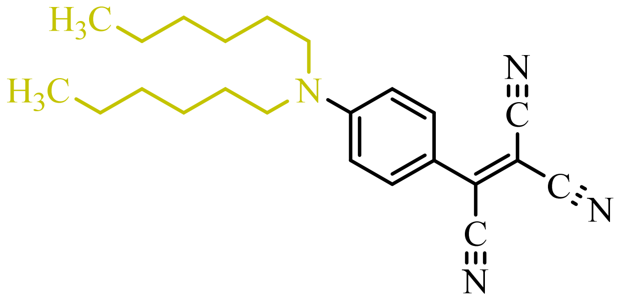

Pink solid; mp 93-94 °C (ethyl acetate); υ_{max}/cm^{-1} (KBr): 2214 (C≡N), 1612 (aromatic C=C) and 1384 (C-N); ^{1}H NMR (300 MHz, $CDCl_3$, Me_4Si) δ_H 8.05 (2H, d, J = 9.3 Hz, ArH), 6.68 (2H, d, J = 9.3 Hz, ArH), 3.42 (4H, t, J = 7.5 Hz, 2xCH_2), 1.59-1.65 (3H, m, CH_3), 1.25-1.35 (13H, m), 0.91 (6H, t, J = 6.6 Hz); ^{13}C NMR (125 MHz, $CDCl_3$) δ_c 14.0, 22.6, 26.7, 27.4, 31.5, 51.7, 75.9, 112.4, 114.1, 114.4, 114.6, 117.3, 133.2, 136.7, 153.7; MS = 363.7 (M+H).

2-[4-(1-Pyrrolidinyl)phenyl]-1,1,2-ethenetricarbonitrile (34g)

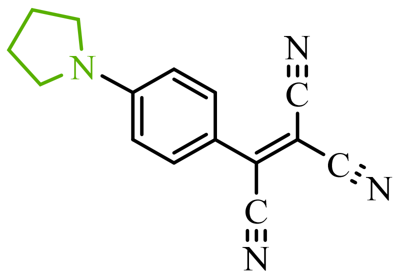

Blue solid; mp 197-199 °C (ethyl acetate); υ_{max}/cm^{-1} (KBr): 2209 (C≡N), 1608 (aromatic C=C) and 1384 (C-N); ^{1}H NMR (300 MHz, $CDCl_3$, Me_4Si) δ_H 8.08 (2H, d, *J* = 9 Hz, Ar*H*), 6.64 (2H, d, *J* = 9 Hz, Ar*H*), 3.53 (4H, t, *J* = 6.6 Hz, 2xC*H*2), 2.13 (4H, m, *J* = 6.6 Hz, 2xC*H*2); MS = 249.6 (M+H).

2-[4-(Phenylamino)phenyl]-1,1,2-ethenetricarbonitrile (34h)

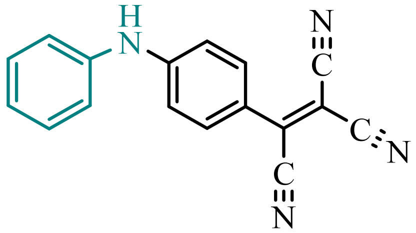

Green solid; mp 188-190 °C (ethyl acetate); υ_{max}/cm^{-1} (KBr): 3311 (N-H), 2219 (C≡N), 1609 (aromatic C=C) and 1384 (C-N); ^{1}H NMR (300 MHz, $CDCl_3$, Me_4Si) δ_H 8.06 (2H, t, *J* = 9 Hz, Ar*H*), 7.44 (2H, d, *J* = 8.7 Hz, Ar*H*), 7.24-7.29 (3H, m, Ar*H*), 7.01 (2H, t, *J* = 9 Hz, Ar*H*), 6.70 (1H, bs, N*H*); MS = 269.4 (M-H).

1.1. Indole derivatives 34b-g [22]

2-(2-Methyl-1H-indol-3-yl)-1,1,2-ethenetricarbonitrile (36i)

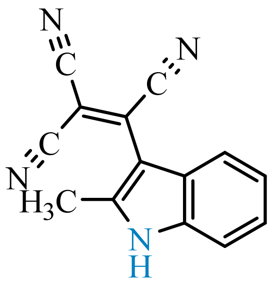

Red solid; mp 183-185 °C (ethyl acetate); υ_{max}/cm^{-1} (KBr): 3283 (N-H), 2235 (C≡N), 1620 (aromatic C=C) and 1384 (C-N); ^{1}H NMR (300 MHz, $CDCl_3$, Me_4Si) δ_H 9.01 (bs, 1H, NH), 7.78-7.81 (1H, m, Ar*H*),7.27-7.42 (3H, m, Ar*H*), 2.70 (3H, s, C*H*3); MS = 231.3 (M-H).

2-(2-Methyl-1-propyl-1H-indol-3-yl)ethene-1,1,2-tricarbonitrile (36j)

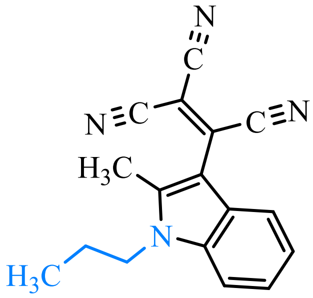

Golden red solid; mp 177-178 °C (ethyl acetate); υ_{max}/cm^{-1} (KBr): 2214 (C≡N), 1617 (aromatic C=C) and 1384 (C-N); ^{1}H NMR (300 MHz, $CDCl_3$, Me_4Si) δ_H 7.80-7.85 (1H, m, Ar*H*), 7.34-7.41 (3H, m, Ar*H*), 4.16 (3H, t, J = 7.5), 2.62 (3H, s, CH_3), 1.81-1.93 (2H, m, J = 7.5), 1.03 (2H, t, J = 7.5, CH_2); ^{13}C NMR (125 MHz, $CDCl_3$) δ_c 11.4, 14.4, 22.8, 46.2, 84.1, 108.7, 110.9, 112.7, 113.0, 114.1, 120.3, 123.6, 124.7, 124.8, 134.2, 137.1, 145.5; MS = 275.6 (M+H).

2-(1-(3-Chloropropyl)-2-methyl-1H-indol-3-yl)ethene-1,1,2-tricarbonitrile (36k)

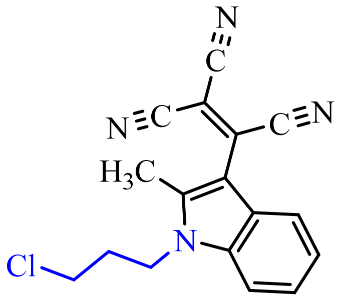

Golden orange solid; mp 141-143 °C (ethyl acetate); υ_{max}/cm^{-1} (KBr): 2218 (C≡N), 1616 (aromatic C=C) and 1384 (C-N); ^{1}H NMR (300 MHz, $CDCl_3$, Me_4Si) δ_H 7.85-7.87 (1H, m, Ar*H*), 7.48-7.49 (1H, m, Ar*H*), 7.42-7.43 (2H, m, Ar*H*), 4.45 (2H, t, J = 3.6 Hz, CH_2), 3.63 (2H, t, J = 3.6 Hz, CH_2), 2.7 (3H, s, CH_3), 2.33 (2H, q, J = 3.6 Hz); MS = 307.3 (M-H).

2-(1-Butyl-2-methyl-1H-indol-3-yl)ethene-1,1,2-tricarbonitrile (36l)

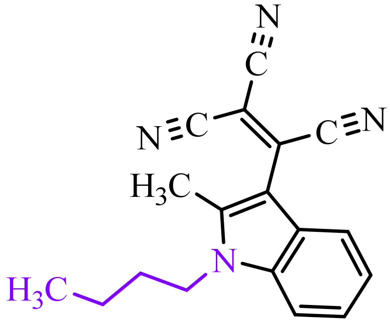

Golden orange solid; mp 138-140 °C (ethyl acetate); υ_{max}/cm^{-1} (KBr): 2216 (C≡N), 1617 (aromatic C=C) and 1384 (C-N); ^{1}H NMR (300 MHz, $CDCl_3$, Me_4Si) δ_H 7.35-7.39 (3H, m, Ar*H*), 7.81-7.84 (1H, m, Ar*H*), 4.18 (3H, t, *J* = 7.2 Hz, C*H*3), 2.62 (3H, s, C*H*3), 1.75-1.85 (2H, m, *J* = 7.2 Hz), 1.38-1.51 (2H, m, C*H*2), 1.00 (2H, t, *J* = 7.2, C*H*2); ^{13}C NMR (125 MHz, $CDCl_3$) δ_c 13.7, 14.4, 20.2, 31.5, 44.5, 84.1, 108.7, 110.8, 112.7, 113.0, 114.1, 120.4, 123.6, 124.7, 124.8, 134.2, 137.0, 145.3; MS = 289.6 (M+H).

2. Derivatives of dimethylaminonitrosoamine [26]

4-(Dimethylamino)phenyliminolmalononitrile N-oxide (40).

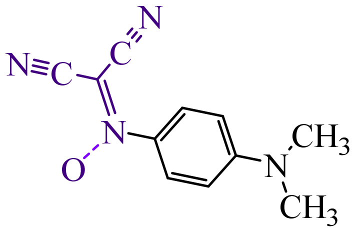

Dark violet crystals, m.p. 141—143 °C ^{14}N NMR (acetone-d_6), δ_N -44 (N=N(O), $\Delta\nu_{1/2}$ = 50 Hz), -100 (CN, $\Delta\nu_{1/2}$ = 600 Hz), -320 (Me_2N, $\Delta\nu_{1/2}$ = 500 Hz). UV (hexane), L_{max}/nm: 465 (lgz 3.57. UV (MeCN), λ_{max}/nm 495. ^{1}H NMR ($CDCl_3$) δ_H 7.74 (2H, d, *J* =7.1 Hz, ArH); 6.65 (2H, d, *J* =7.1 Hz, ArH); 3.15 (6H, s, 2xCH_3); ^{13}C NMR ($CDCl_3$) δ_c 154.4, 134.3,125.7, 110.9, 110.7, 93.2, 40.4.

4-(Dimethylamino)phenylimino]malononitrile (41).

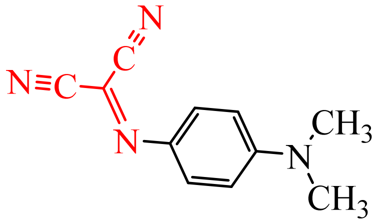

Red crystals, m.p. 169—171 °C ^{14}N NMR (acetone-d_6), δ_N - 10 (CN, $\Delta\nu_{1/2}$= 700 Hz), -310 (Me_2N, $\Delta\nu_{1/2}$ = 600 Hz). ^{1}H NMR (acetone-d_6) δ_H 7.79 (2H, d, *J* =9.6 Hz, ArH); 6.92 (2H, d, *J* =9.6 Hz, ArH); 3.25 (6H, s, 2xCH_3); ^{13}C NMR (acetone-d_6) δ_c 134.5,129.2, 113.0, 112.3, 154.6, 93.4, 40.0.

3. Shiff`s base derivatives [8]

2-{4-[2-Benzylidenehydrazino]phenyl}ethylene-1,1,2-tricarbonitrile (44a)

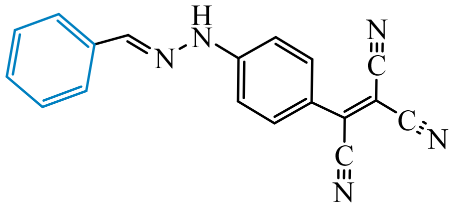

m.p. 285-288 °C, yield 76%, UV-Vis (acetone): λmax (nm) 525, 333; FTIR ν(cm^{-1}): 3262 (sec. NH), 2212 (CN), 1609 (C=N), 1337 (C-N); ^{1}H NMR (DMSO-d_6): 11.84 (s, 1H, NH; cancelled with D_2O), 8.14 (s, 1H, CH=N), 7.42-8.01 (m, 9H,Ar-H). ^{13}C NMR (DMSO-d_6): 162.29 (CH=N), 151.19 (12C-Ar), 144.48 (C=C), 137.3, 134.2, 132.7, 2x129.9, 2x128.8, 126.6, 119.4, 3x114.8 (3CN), 113.9, 112.95, 79.56.

2-{4-[2-(1-Naphthylmethylene)hydrazino]phenyl}ethylene-1,1,2-tricarbonitrile (44b)

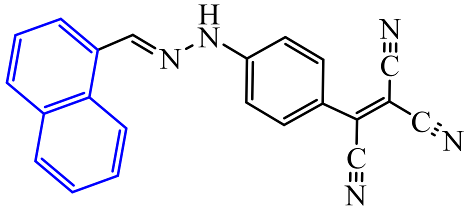

m.p. 268-270 °C, yield 87%; UV-Vis (acetone): λmax (nm) 529, 353; FTIR ν(cm^{-1}): 3208 (sec. NH), 2212 (CN), 1611 (C=N), 1341 (C-N); ^{1}H NMR ($CDCl_3$): δ_H 11.95 (s, 1H, NH), 8.76(s, 1H, CH=N), 7.37-8.05 (m, 11H, Ar-H); ^{13}C NMR (DMSO-d6): δ_C 162.27 (CH=N), 144.48(C=C), 137.32(16C-Ar), 133.57, 132.84, 130.44, 129.62, 129.41, 128.88, 127.68, 127.52, 2x126.30, 125.61, 123.96, 2x119.52, 3x114.84 (3CN), 113.94, 78.15.

2-{4-[2-(9-Anthrylmethylene)hydrazino]phenyl}ethylene-1,1,2-tricarbonitrile (44c)

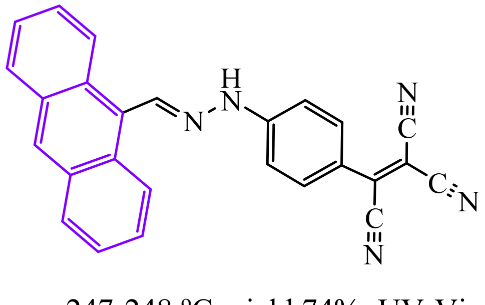

m.p. 247-248 °C, yield 74%; UV-Vis (acetone): λmax (nm) 540, 407; FTIR ν(cm^{-1}): 3211 (sec. NH), 2209 (CN), 1603 (C=N), 1334 (C-N); ^{1}H NMR (DMSO-d6): δ_H 10.80 (s, 1H, NH), 8.30(s, 1H, CH=N), 6.80-8.20 (m, 13H, Ar-H). ^{13}C NMR (DMSO-d6): δ_C 162.50 (C=N), 143.40, (C=C), 135.03 (20C-Ar), 132.87, 132.31, 130.90, 129.70, 129.23, 128.96, 128.64, 128.17, 127.38, 126.87, 125.75, 125.48, 125.31, 124.90, 124.64, 124.18, 120.22, 118.93, 3x112.49 (3CN), 111.86, 79.0.

4. 6,9-Diphenyl-7,8-dihydro-1H,4H-benzocyclooctene-2,2,3,3-tetracarbonitrile [27]

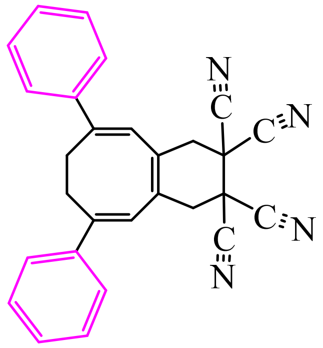

yield, 84%. ^{1}H NMR (400 MHz, $CDCl_3$, 258C, TMS): δ_H 7.34–7.30 (m, 10H), 5.94 (s, 2H), 3.28 (s, 4H), 2.88 ppm (s, 4H); ^{13}C NMR (100 MHz, CDCl3): δ_C 147.3, 141.9, 128.6, 128.3, 126.2, 124.2, 124.0, 110.6, 37.9, 36.6, 30.4 ppm; IR (film): n˜ = 3055, 1941 cm^{-1}; HRMS (EI) calcd for C28H20N4M+ 412.1688; found 412.1689.

5. Arylaminoquinazolines [29]

4-(p-Tolylamino)quinazoline-2-carbonitrile (50b)

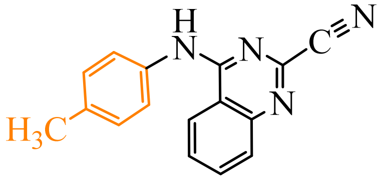

(255.0 mg, 98%) as colorless needles, mp (hotstage) 208-209 °C (from $CHCl_3$), IR ν_{max}/cm^{-1} (KBr): 3389w (NH), 3048w (Ar CH), 2953w, 2918w and 2855w (Alk CH), 2243w (C≡N), 1618s, 1607s, 1574s, 1566s, 1530s, 1495s, 1454w, 1423m, 1369s, 1319w, 1304w, 1267w, 1258w, 1234w, 1223w, 1217w, 1134w, 1090w, 997w, 864w, 810m, 799m, 790m, 768s; ^{1}H NMR (500 MHz; DMSO-d_6): δ_H 10.25 (1H, br s, NH), 8.60 (1H, d, J 8.0, Ar H), 7.97 (1H, ddd, J 7.8, 7.8, 1.0, Ar H), 7.86 (1H, d, J 8.0, Ar H), 7.79 (1H, ddd, J 7.5, 7.5, 1.0, Ar H), 7.60 (2H, d, J 8.5, Ar H), 7.25 (2H, d, J 8.5, Ar H), 2.33 (3H, s, CH3); ^{13}C NMR (75 MHz; DMSO-d6): δ_C 158.6 (s), 148.9 (s), 140.2 (s), 135.4 (s), 134.5 (s), 134.4 (d), 129.0 (d), 128.2 (d), 128.0 (d) 123.6 (d), 123.5 (d), 117.0 (s), 115.6 (s), 20.6 (q, CH3); m/z (MALDI-TOF) 262 (MH+ +1, 19%), 261 (MH+ , 100), 236 (2).

4-[(4-Methoxyphenyl)amino]quinazoline-2-carbonitrile (50c)

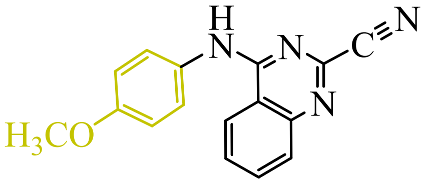

(268.4 mg, 97%) as pale yellow needles, mp (hotstage) 198-199 °C, IR ν_{max}/cm^{-1} (KBr): 3350m (NH), 3075w and 3049w (Ar CH), 2953w (Alk CH), 2249w (C≡N), 1612w, 1600m, 1582s, 1564w, 1558w, 1514s, 1489m, 1456w, 1429w, 1408w, 1373m, 1358w, 1314m, 1296m, 1261m, 1253m, 1236m, 1225w, 1186w, 1171m, 1128w, 1111w, 1094w, 1038m, 995w, 868w, 860w, 820w, 799w, 787w, 768s, 758m; ^{1}H NMR (500 MHz; DMSO-d6): δ_H 10.25 (1H, br s, NH), 8.57 (1H, d, J 8.5, Ar H), 7.96 (1H, dd, J 7.8, 7.8, Ar H), 7.86 (1H, d, J 8.0, Ar H), 7.78 (1H, dd, J 7.5, 7.5, Ar H), 7.61 (2H, d, J 7.8, Ar H), 7.03 (2H, d, J 7.5, Ar H), 3.79 (3H, s, OCH3); ^{13}C NMR (125 MHz; DMSO-d_6): δ_C 158.6 (s), 156.9 (s), 148.8 (s), 140.3 (s), 134.4 (d), 130.7 (s), 129.0 (d), 128.1 (d), 125.3 (d), 123.4 (d), 117.1 (s), 115.6 (s), 114.0 (d), 55.4 (q, OCH3); m/z (EI) 278 (MH+ +1, 17%), 277 (MH+ , 100), 252 (2), 215 (1).

4-[(4-Fluorophenyl)amino]quinazoline-2-carbonitrile (50d)

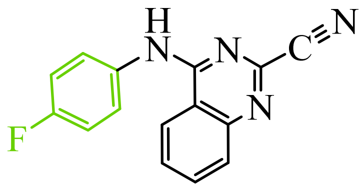

(258.8 mg, 98%) as pale yellow needles, IR λ_{max}(DCM)/nm 258 inf (log ε 4.22), 268 inf (4.20), 282 inf (4.15), 332 (4.35); ν_{max}/cm^{-1} (KBr): 3385w (NH), 3059w and 3034w (Ar CH), 2247w (C≡N), 1616m, 1607m, 1570s, 1558m, 1533m, 1506w, 1495s, 1456w, 1449w, 1425w, 1369m, 1321w, 1256w, 1246w, 1234w, 1217w, 1161w, 1130w, 1105w, 1034w, 976w, 955w, 897w, 864w, 831w, 795w, 789w, 766s, 745s; ^{1}H NMR (500 MHz; DMSO-d_6): δ_H 10.34 (1H, br s, NH), 8.59 (1H, d, J 8.5, Ar H), 7.99 (1H, dd, J 7.8, 7.8, Ar H), 7.89 (1H, d, J 8.5, Ar H), 7.81 (1H, dd, J 7.8, 7.8, Ar H), 7.79-7.74 (2H, m, Ar H), 7.33-7.28 (2H, m, Ar H); ^{13}C NMR (125 MHz; DMSO-d_6); δ_C 159.3 (d, 1 JCF 241.3), 158.5 (s), 148.8 (s), 140.0 (s), 134.4 (d), 134.2 (s), 129.0 (d), 128.1 (d), 125.4 (d, 3 JCF 8.3), 123.4 (d), 116.9 (s), 115.41 (d, 2 JCF 22.3), 115.44 (s); m/z (EI) 265 (MH+ , 15%), 248 (55), 247 (100), 236 (7).

4-[(4-Chlorophenyl)amino]quinazoline-2-carbonitrile (50e)

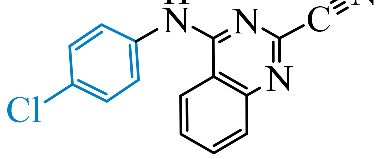

(264.8 mg, 95%) as colorless needles, mp 230-231 °C; IR ν_{max}/cm^{-1} (KBr): 3348w (NH), 3063w (Ar CH), 2251w (C≡N), 1614m, 1601m, 1574s, 1564m, 1526s, 1497m, 1487s, 1456w, 1425m, 1400w, 1368m, 1359w, 1314w, 1258w, 1223w, 1179w, 1126w, 1098w, 1092w, 1016w, 993w, 864w, 854w, 816m, 787m, 766s, 752w; ^{1}H NMR (500 MHz; DMSO-d_6): δ_H

10.37 (1H, br s, NH), 8.60 (1H, d, J 8.0, Ar H), 7.99 (1H, ddd, J 7.8, 7.8, 1.0, Ar H), 7.89 (1H, d, J 8.0, Ar H), 7.84-7.79 (3H, m, Ar H), 7.51 (2H, d, J 7.5, Ar H); ^{13}C NMR (125 MHz; DMSO-d_6): δ_C 158.4 (s), 149.0 (s), 139.9 (s), 137.2 (s), 134.6 (d), 129.2 (d), 128.8 (s), 128.7 (d), 128.2 (d), 124.9 (d), 123.5 (d), 117.0 (s), 115.7 (s); m/z (MALDI-TOF) 283 (MH+ +2, 25%), 282 (MH+ +1, 9), 281 (MH+ , 100), 153 (7), 130 (3).

4-[(4-Bromophenyl)amino]quinazoline-2-carbonitrile (50f)

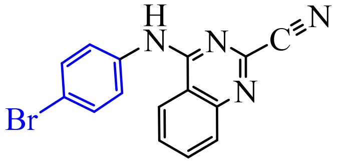

(314.7 mg, 97%) as pale yellow needles, mp 234.5-235 ºC (from $CHCl_3$), ν_{max}/cm^{-1} (KBr): 3352w (NH), 3063w (Ar CH), 2255w (C≡N), 1616m, 1603m, 1570s, 1562m, 1522s, 1487s, 1456w, 1422m, 1398w, 1368m, 1354w, 1315m, 1296w, 1258w, 1236w, 1217w, 1182w, 1128w, 1076m, 1009w, 993m, 951w, 937w, 870w, 845w, 820s, 791m, 764s; ^{1}H NMR (500 MHz; DMSO-d_6): δ_H 10.35 (1H, br s, Ar H), 8.60 (1H, d, J 8.0, Ar H), 8.00 (1H, dd, J 7.8, 7.8, Ar H), 7.90 (1H, d, J 8.5, Ar H), 7.82 (1H, dd, J 7.5, 7.5, Ar H), 7.75 (2H, d, J 8.5, Ar H), 7.64 (2H, d, J 7.5, Ar H); ^{13}C NMR (125 MHz; DMSO-d_6): δ_C 158.4 (s), 149.0 (s), 139.9 (s), 137.6 (s), 134.6 (d), 131.7 (d), 129.3 (d), 128.3 (d), 125.2 (d), 123.5 (d), 117.0 (s), 115.7 (s); m/z (MALDI-TOF) 327 (MH+ +2, 28%), 325 (MH+ , 33), 309 (4), 308 (19), 307 (100), 282 (3), 153 (30).

4-[(3,4-Dimethoxyphenyl)amino]quinazoline-2-carbonitrile (50g)

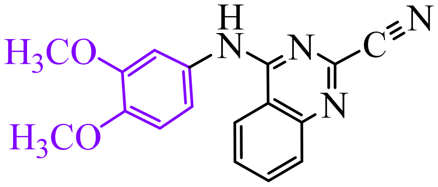

(286.0 mg, 93%) as pale yellow plates, mp (hotstage) 202-203 ºC (from $CHCl_3$); ν_{max}/cm^{-1} (KBr): 3375w (NH), 3017w (Ar CH), 2937w and 2837w (Alk CH), 2247w (C≡N), 1622m, 1609w, 1574s, 1530m, 1518m, 1499s, 1464m, 1429w, 1414w, 1373m, 1323w, 1307w, 1265w, 1279w, 1236w, 1223s, 1202m, 1175w, 1165w, 1144m, 1090w, 1042w, 1028s, 999w, 955w, 870w, 843w, 800m, 792s, 772m, 761s, 746s; ^{1}H NMR (500 MHz; DMSO-d_6): δ_H 10.23 (1H, br s, NH), 8.60 (1H, d, J 8.0, Ar H), 7.97 (1H, dd, J 7.8, 7.8, Ar H), 7.87 (1H, d, J 7.5, Ar H), 7.80 (1H, dd, J 7.8, 7.8, Ar H), 7.38 (1H, d, J 2.5, Ar H), 7.31 (1H, dd, J 9.0, 2.3, Ar H), 7.04 (1H, d, J 8.5, Ar H), 3.79 (6H, s, 2 × OCH3); ^{13}C NMR (125 MHz; DMSO-d_6): δ_C 158.5 (s), 148.9 (s), 148.6 (s), 146.5 (s), 140.2 (s), 134.4 (d), 131.1 (s), 129.0 (d), 128.2 (d), 123.4 (d),

117.0 (s), 115.8 (d), 115.6 (s), 111.8 (d), 108.5 (d), 55.8 (q, OCH3), 55.7 (q, OCH3); m/z (MALDI-TOF) 308 (MH+ +1, 13%), 307 (MH+ , 100), 306 (M+ , 8).

6. (E)-3-(5-((2-cyanoquinazolin-4-yl)(methyl)amino)-2- methoxyphenyl)-N-hydroxyacrylamide (62) [30]

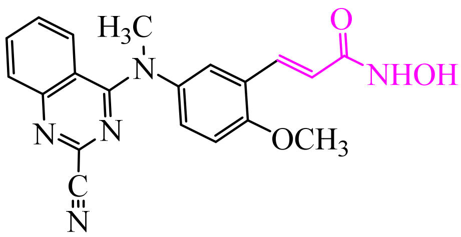

Colorless oil. ^{1}H NMR (300 MHz, DMSO-d6) δ 7.79 (m, 2H), 7.64–7.55 (m, 2H), 7.42–7.25 (m, 2H), 7.17 (d, J = 8.7 Hz, 1H), 7.00 (d, J = 8.4 Hz, 1H), 6.51 (d, J = 15.8 Hz, 1H), 3.91 (s, 3H), 3.56 (s, 3H). ^{13}C NMR (75 MHz, DMSO-d6) δ 162.7 (C), 160.9 (C), 156.5 (C), 150.4 (C), 139.5 (2C), 133.4 (CH), 132.3 (CH), 128.4 (2CH), 127.7 (CH), 126.0 (CH), 125.8 (CH), 124.9 (C), 121.1 (CH), 116.9 (C), 116.2 (C), 113.3 (CH), 56.1 (OCH_3), 42.9 (CH_3). HRMS (ESI+) (M + H)+: m/z calcd for C20H18N5O3 376.1410, found 376.1393. HPLC: 100%

6. 2-Aminobenylamine derivatives

1,4-Dihydroquinazolin-2-carbonitrile (67)

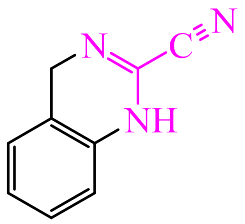

Deep-red powder (yield: 35 mg, 22%), m. p. 121 – 123 ◦C. – IR (film): ν = 3278 (NH), 3135, 3059, 2976, 2864 (CH), 2180 (CN), 1695, 1619, 1575 (C=N, C=C) cm^{-1} . – ^{1}H NMR (400 MHz, CDCl3): δ = 4.73 (s, 2H, CH2), 6.91 (d, 1H, J = 7.41 Hz), 7.09 – 7.13 (m, 2H), 7.20 – 7.24 (m, 1H), 7.31 – 7.37 (m, 1H). – ^{13}C NMR (100 MHz, $CDCl_3$): δ = 42.02 (CH_2), 108.83 (C), 126.01 (CH), 126.41 (CH), 128.12 (C), 128.55 (CH), 128.59 (CH), 129.68 (CH), 147.49 (C). – MS (EI, 70 eV): m/z(%) = 158 (5) [M+1] +, 157 (40) [M] +, 156 (100) [M−1] +, 129 (22) [M−HCN] +, 102 (6), 77 (10). – $C_9H_7N_3$ (157.06): calcd. C 68.78, H 4.49, N 26.74; found C 68.61, H 4.44, N 26.60.

2-(3,4-Dihydroquinazolin-2(1H)-ylidene)malononitrile (68)

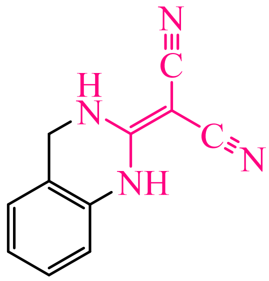

Colorless powder (yield: 147 mg, 75%), m. p. > 240 ◦C. – IR (film): ν = 3272, 3218 (NH), 3168, 3059, 2944, 2870 (CH), 2207, 2175 (CN), 1596, 1552 (C=C) cm^{-1} . – 1H NMR (400 MHz, [D6]DMSO): δ = 4.38 (d, 2H, CH2, J = 1.00 Hz), 7.04 – 7.09 (m, 1H), 7.15 (d, 1H, J = 7.41 Hz), 7.19 – 7.25 (m, 2H), 8.32 (s, 1H, NH), 10.26 (s, 1H, NH). – ^{13}C NMR (100 MHz, [D6]DMSO): δ = 41.12 (CH2), 115.95 (CH), 117.62 (C), 119.16 (C), 124.08 (CH), 125.91 (CH), 128.16 (CH), 133.71 (C), 158.05 (C). – MS (EI, 70 eV): m/z(%) = 197 (7) [M+1] +, 196 (75) [M] +, 195 (100) [M−1] +, 168 (22) [M−HCN] +, 141 (10), 129 (7), 116 (4), 104 (6), 97 (4), 89 (2), 77 (8). – $C_{11}H_8N_4$ (196.07): calcd. C 67.34, H 4.11, N 28.55; found C 67.25, H 4.13, N 28.49.

7. Pyrazoles [47, 48]

3-Amino-4,5-dicyano-1-methylpyrazole (78a) [47]

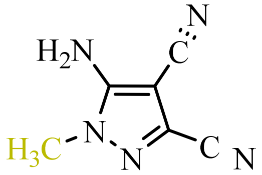

Colorless needles: yield 6.66 g (53%); m.p. 135- 135.5 °C; λ_{max} (EtOH) (pH 1) 302 nm (e 4700) and 239 (9300), λ_{max} 264 (800) and 229 (8100); λ_{max} (EtOH) (pH 7) 302 (4700) and 239 (9300), λ_{max} 263 (800) and 229 (8300); λ_{max}, (EtOH) (pH 11) 298 (3700) and 240 (8400), A,, 267 (2200) and 231 (7700); MS m/e 147, 122, 121, 120, 119, 104, 77, and 76; ir (Nujol) 3440,3350,3220,2955,2920,2850,2245,2225,1630,1550, and 1520 cm-l

5-Amino-1-methyl-1*H*-pyrazole-3,4-dicarbonitrile (73a) [47, 48]

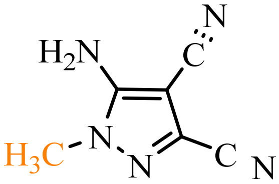

The yield is 91%, m. p. 233-235 °C (MeOH). IR spectrum, ν_{max}/cm^{-1}: 3410, 3341, 3251 (NH2), 2252, 2223 (C≡N), 1662, 1591 (C=C). 1H NMR, δ, ppm.: 7.19 (s, 2H, NH2), 3.61 (s, 3H, CH3). 13C NMR spectrum, δc, ppm: 152.85 (C4), 124.22 (C2), 112.93 (C7N9), 112.90

(C8N10), 76.63 (C3), 36.50 (CH36). Found, %: C 49.15; H 3.54; N 41.31. $C_6H_5N_5$. Calculated, %: C 48.98; H 3.43; N 47.60.

5-Amino-1-(pyridin-2-yl)-1*H*-pyrazole-3,4-dicarbonitrile (3b)

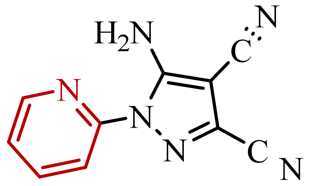

The yield is 89%, m.p. 183-185 °C (EtOH). IR spectrum, ν_{max}/cm^{-1}: 3290, 3200 (NH2), 2227 (C≡N), 1586 (C=C). 1H NMR , δ, ppm.: 8.52 s (2H, NH2), 8.50 – 8.44 m (1H, CH13), 8.13 – 7.94 m (1H, CH15), 7.81 dt („*J* = 8.4, 0.9 Hz, 1H, CH16), 7.55 – 7.33 m (1H, CH14). 13C NMR, δc, ppm: 152.98 (C4), 152.16 (C6), 147.26 (CH13), 140.31 (CH15), 126.39 (C2), 122.72 (CH14), 113.85 (C7N9), 111.76 (C8N10). Found, %: C 57,22; H 3,14; N 39,64. C10H6N6. Calculated, %: C 57.14; H 2.88; N 39.98.

8. 1,2,4-triazolium-3-thiolates [49]

5-(1-Amino-2,2-dicyanovinyl)-4-ethyl-1-phenyl-4H-1,2,4-triazol-1-ium-3- thiolate (88a)

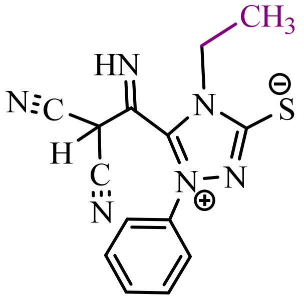

Colorless crystals (0.225 g, 76%), mp = 245–247 °C, MeCN. IR (KBr) m = 3340 (NH2), 2215 (CN), 1645 (C=N), 1588, 1486 (ArAC=C and conjugated cyclic C=N), 1290 (C=S str.), ^{1}H NMR (400 MHz, CDCl3): dH = 10.18 (br s, 2H, NH2), 7.40–7.33 (m, 2H, Ar=CH), 7.21–7.17 (m, 3H, ArAH), 3.81 (q, 2H, J = 7.64, CH2), 1.28 (t, 3H, J = 7.64, CH3). ^{13}C NMR (100 MHz, CDCl3): dc = 171.4 (=C-NH2), 169.8 (triazolium-C-3), 151.7 (triazolium-C-5), 138.9 (Ar-C), 131.2, 128.7, 124.2 (Ar=CH), 110.8 (CN), 60.4 (C(CN)2), 37.2 (CH2), 15.2 (CH3). MS (EI): m/z = 296 (M+, 100), 253(26), 147 (34), 105 (51), 77 (64). Anal. Calcd for C14H12N6S (296.35): C, 56.74; H, 4.08; N, 28.36; S, 10.82. Found: C, 56.87; H, 3.96; N, 28.29; S, 10.95.

4-Allyl-5-(1-amino-2,2-dicyanovinyl)-1-phenyl-4H-1,2,4-triazol-1-ium-3-thiolate (88b)

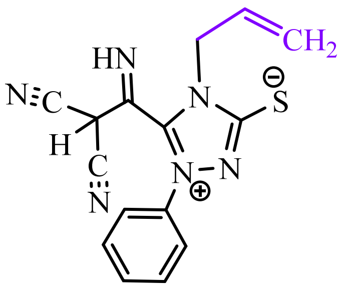

Colorless crystals (0.219 g, 71%), mp = 190–191 C, MeCN. IR (KBr) m = 3325 (NH2), 2220 (CN), 1648 (C=N), 1590, 1495 (Ar-C=C and conjugated cyclic C=N), 1292 (C-S$^-$ str.), 1 H NMR (400 MHz, CDCl3): dH = 10.28 (br s, 2H, NH2), 7.34–7.22 (m, 2H, Ar-H), 7.19–7.10 (m, 3H, Ar-H), 5.90–5.87 (m, 1H, allyl-CH=), 5.28–5.23 (m, 2H, allyl-CH2-), 4.63–4.62 (m, 2H, allyl-CH2N). 13C NMR (100 MHz, CDCl3): dc = 171.2 (=C-NH2), 169.8 (triazolium-C-3), 151.1 (triazolium-C-5), 138.6 (Ar-C), 133.5 (allyl-CH=), 131.3, 129.4, 128.5 (Ar-CH), 121.4 (allyl-CH2), 110.9 (CN), 60.5 (C (CN)2), 49.7 (allyl-CH2N). MS (EI): m/z = 308 (M+ , 100), 253(32), 209 (18), 203 (11), 105 (28), 99 (34), 77 (42), 41 (26). Anal. Calcd for C15H12N6S (308.36): C, 58.43; H, 3.92; N, 27.25; S, 10.40. Found: C, 58.29; H, 4.06; N, 27.41; S, 10.27.

5-(1-Amino-2,2-dicyanovinyl)-1,4-diphenyl-4H-1,2,4-triazol-1-ium-3-thiolate (88c)

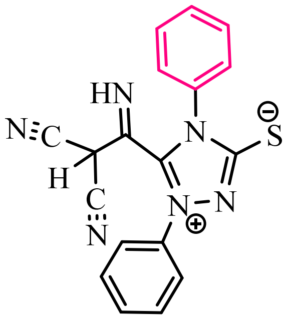

Colorless crystals (0.230 g, 67%), mp = 300–302 C, MeCN. IR (KBr) m = 3330 (NH2), 2210 (CN), 1688 (C=N), 1595, 1492 (Ar-C=C and conjugated cyclic C=N), 1286 (C-S str.), ^{1}H NMR (400 MHz, $CDCl_3$): dH = 10.34 (br s, 2H, NH2), 7.51–7.44 (m, 2H, Ar-H), 7.42–7.35 (m, 6H, Ar-H), 7.33–7.28 (m, 2H, Ar-H). 13C NMR (100 MHz, $CDCl_3$): dc = 171.2 (=C-NH2), 169.7 (triazolium-C3), 151.4 (triazolium-C-5), 138.4, 131.9 (Ar-C), 131.6, 131.2, 129.7, 129.5, 128.6, 128.5 (Ar-CH), 111.2 (CN), 60.1 (C (CN)2). MS (EI): m/z = 344 (M+ , 100), 209(37), 135 (71), 105 (28), 77 (53). Anal. Calcd for C18H12N6S (344.39): C, 62.77; H, 3.51; N, 24.40; S, 9.31. Found: C, 62.91; H, 3.38; N, 24.56; S, 9.19.

9. (Z)-N-[4-Amino-2-(benzylimino)-5-cyanothiazol-3(2H)-yl]- picolinamide (98a)

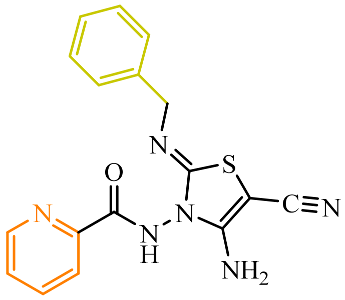

(C17H14N6OS) Yellow crystals (acetonitrile); Rf = 0.27; m.p.: 124–125 °C; yield: 276 mg (79%); IR (KBr): 휧 =3305 (NH2), 3187 (NH), 3082 (Ar–CH), 2189 (C≡N), 1701 (C=O), 1623 (C=N), 1585 (Ar–C=C) cm−1; 1 H NMR (400 MHz, DMSO-d6): δ=11.30 (s, 1H, amideNH), 8.76–8.66 (m, 1H, Pyr-H), 8.10–7.99 (m, 2H, Pyr-H), 7.70–7.66 (m, 1H, Pyr-H), 7.45 (s, 2H, NH2 attached to thiazole), 7.35–7.12 (m, 5H, Ph-H), 4.21 (s, 2H, CH2Ph) ppm; 13C NMR (100 MHz, DMSO-d6): δ=163.88 (amideCO), 161.65 (thiazole-C4), 153.6 (thiazole-C2), 150.6 (PyrC), 148.8 (Pyr-CH), 139.5 (Pyr-CH), 138.0 (Ph-C), 128.3 (Ph-2CH), 127.7 (Ph-CH), 127.3 (Ph-2CH), 126.7, 123.0 (Pyr-CH), 116.8 (C≡N), 57.7 (CH2Ph), 55.83 (thiazole-C5) ppm; MS (FAB): m/z (%)=350 (M+, 100).

(Z)-N-[2-(Allylimino)-4-amino-5-cyanothiazol-3(2H)-yl]pico- linamide (98b)

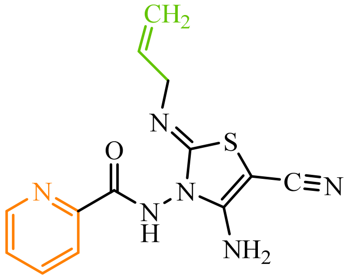

Yellow crystals (acetonitrile); Rf=0.29; m.p.: 170–171 °C; yield: 213 mg (71%); IR (KBr): 휧 =3284 (NH2), 3185 (NH), 3066 (Ar–CH), 2191 (C≡N), 1700 (C=O), 1630 (C=N), 1589 (Ar–C=C) cm−1; 1 H NMR (400 MHz, DMSO-d6): δ=11.25 (s, 1H, amide- NH), 8.83– 8.62 (m, 1H, Pyr-H), 8.21–7.92 (m, 2H, Pyr-H), 7.72–7.66 (m, 1H, Pyr-H), 7.43 (s, 2H, NH2 attached to thiazole), 5.85– 5.76 (m, 1H, allyl-CH=), 5.40–4.87 (m, 2H, allyl-CH2=), 3.66–3.62 (m, 2H, allyl-CH2N) ppm; 13C NMR (100 MHz, DMSO-d6): δ = 164.1 (amide-CO), 153.6 (thiazole-C4) 150.3 (Pyr-C), 148.9 (Pyr-CH), 148.8 (thiazole-C2), 138.1 (Pyr-CH), 135.3 (allyl-CH=), 127.8, 123.1 (Pyr-CH), 116.9 (allyl-CH2=), 115.4 (C≡N), 55.0 (thiazole-C5), 43.6(allylCH2) ppm; MS (FAB): m/z (%)=300 (M+, 100).

(Z)-N-[4-Amino-2-(benzylimino)-5-cyanothiazol-3(2H)-yl]- furan-2-carboxamide (98c)

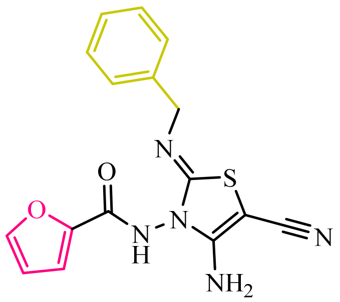

Orange crystals (acetonitrile); Rf=0.44; m.p.: 180–181 °C; yield: 250 mg (74%); IR (KBr): 흸 =3310 (NH_2), 3191 (NH), 3045 (Ar– CH), 2190 (C≡N), 1695 (C=O), 1627 (C=N), 1582 (Ar–C=C) cm^{-1}; ^1H NMR (400 MHz, DMSO-d6): δ=11.10 (s, 1H, amide-NH), 7.98–7.88 (m, 1H, furan-H), 7.75 (s, 2H, NH_2 attached to thiazole), 7.69–7.30 (m, 5H, Ph-H), 7.28–7.10 (m, 2H, furan-H), 4.32 (s, 2H, CH_2Ph) ppm; ^{13}C NMR (100 MHz, DMSO-d6): δ=157.3 (amide-CO), 153.3 (thiazole-C4), 145.5 (furan-C2), 139.2 (furan-CH), 146.3 (thiazole-C2), 139.0 (Ph-C), 128.1, 127.1 (Ph-2CH), 126.5 (Ph-CH), 116.4 (C≡N), 116.1, 113.1 (furan-CH), 57.6 (thiazole-C5), 55.8 (CH_2Ph) ppm; MS (FAB): m/z (%)=339 (M+, 100).

(Z)-N-[2-(Allylimino)-4-amino-5-cyanothiazol-3(2H)-yl]- furan-2-carboxamide (98d)

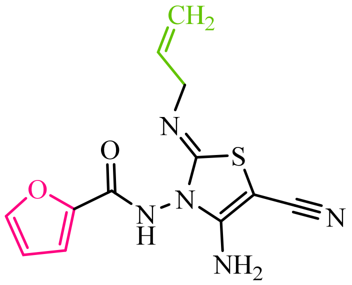

Yellow crystals (acetonitrile); Rf=0.42; m.p.: 130–131 °C; yield: 196 mg (68%); IR (KBr): 흸 =3305 (NH_2), 3181 (NH), 3060 (Ar– CH), 2195 (C≡N), 1700 (C=O), 1623 (C=N), 1590 (Ar–C=C) cm^{-1}; ^1H NMR (400 MHz, DMSO-d6): δ=11.20 (s, 1H, amide-NH), 8.28–8.02 (m, 1H, furan-H), 7.58–7.30 (m, 2H, furan-H), 7.25 (s, 2H, NH_2 attached to thiazole), 5.95– 5.73 (m, 1H, allyl-CH=), 5.47–4.80 (m, 2H, allyl-CH_2=), 3.96–3.61 (m, 2H, allyl-CH_2) ppm; ^{13}C NMR (100 MHz, DMSO-d6): δ = 158.3 (amide-CO), 154.3 (thiazole-C4), 145.3 (furan-C2), 139.3 (furan-CH), 147.3 (thiazole-C2), 133.3 (allyl-CH=), 117.2 (allyl-CH_2=), 116.7 (C≡N), 116.2, 114.1 (furan-CH), 56.8 (thiazole-C5), 48.6 (allyl-CH_2) ppm; MS (FAB): m/z (%)=289 (M+, 50).

(Z)-N-[4-Amino-2-(benzylimino)-5-cyanothiazol-3(2H)-yl]- thiophene-2-carboxamide (98e)

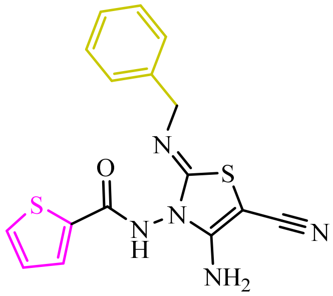

Orange crystals (acetonitrile); Rf=0.51; m.p.: 140–141 °C; yield: 273 mg (77%); IR (KBr): 휈 =3308 (NH2), 3171 (NH), 3070 (Ar–CH), 2186 (C≡N), 1690 (C=O), 1630 (C=N), 1562 (Ar–C=C) cm−1; 1 H NMR (400 MHz, DMSO-d6): δ=11.25 (s, 1H, amide-NH), 8.01–7.78 (m, 3H, thiophene-H), 7.55 (s, 2H, NH2 attached to thiazole), 7.42–7.02 (m, 5H, Ph-H), 4.29 (s, 2H, CH2Ph) ppm; 13C NMR (100 MHz, DMSO-d6): δ=161.1 (amide CO), 153.3 (thiazole-C4), 150.6 (thiazoleC2), 139.3 (thiophene-C2), 135.9 (Ph-C), 132.6, 130.5 (thiophene-CH), 128.1, 127.1 (Ph-2CH), 127.0 (Ph-CH), 126.5 (thiophene-CH), 116.4 (C≡N), 63.4 (thiazole-C5), 55.8 (CH2Ph) ppm; MS (FAB): m/z (%)=355 (M+, 80).

(Z)-N-[2-(Allylimino)-4-amino-5-cyanothiazol-3(2H)-yl]thio- phene-2-carboxamide (98f)

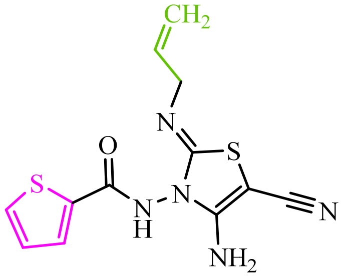

Yellowish orange crystals (acetonitrile); Rf=0.53; m.p.: 152–153 °C; yield: 213 mg (70%); IR (KBr): 휈 =3295 (NH2), 3180 (NH), 3058 (Ar–CH), 2185 (C≡N), 1690 (C=O), 1625 (C=N), 1526 (Ar–C=C) cm−1; 1 H NMR (400 MHz, DMSO-d6): δ=11.20 (br, 1H, amide-NH), 8.10–7.82 (m, 3H, thiophene-H), 7.55 (s, 2H, NH2 attached to thiazole), 5.90–5.74 (m, 1H, allylCH=), 5.21–4.99 (m, 2H, allyl-CH2=), 3.81–3.68 (m, 2H, allyl-CH2N) ppm; 13C NMR (100 MHz, DMSO-d6): δ=160.1 (amide-CO), 154.4 (thiazole-C4), 138.3 (thiazoleC2), 137.2 (thiophene-C2), 133.7, 132.2 (thiophene-CH), 131.3 (allyl-CH=), 129.3 (thiophene-CH), 117.4 (allylCH2=), 114.6 (C≡N), 58.3 (thiazole-C5), 49.6 (allyl-CH2N) ppm; MS (FAB): m/z (%)=305 (M+, 60).

9. 5-Oxo-4,5-dihydro-1H-benzo[e][1,2,4]triazepine-2,2(3H)-dicarbonitrile (101). [51]

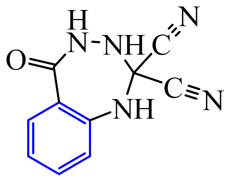

Grey powder; yield (65%); m.p. = 340 °C (decom.); ^{1}H NMR (400 MHz, d6-DMSO): δ 8.20 (d, 1H, NH, J = 7.2 Hz), 8.11 (d, 1H, NH, J = 7.2 Hz), 7.93 (s, 1 H, NH), 7.23–7.04 (m, 4H, ArH) ppm; IR (KBr): ν_{max} = 3278–3190, 2198, 1651, 1515 cm−1 ; MS (EI): m/z (%) = 211 (M-2H, 11), 205 (12), 180 (13), 149 (16), 127 (28), 104 (23), 78 (23). Anal. Calcd for C10H7N5O, (213.20): C, 56.34; H, 3.31; N, 32.85. Found: C, 56.12; H, 3.27; N, 32.76%.

10.

10. p-Methoxyphenyliminomalononitrile (107a) [55]

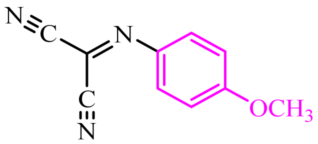

(0.1 58 g, 85%), m.p. 97 "C (orange needles from hexane); $\nu_{max.}$ (KBr): 2 220 (m, CN) and 2 200 cm-' (m, CN); G(CDCI,) 3.92 (s, 3 H), 7.00 (d, J9 Hz, 2 H), and 7.74 (d, J9 Hz, 2 H) (Found: C, 64.8; H, 3.7; N, 22.8. C,,H,N,O requires C, 64.9; H, 3.8; N, 22.7%).

11. Hydrazonomalononitriles [60]

2-[(3-Chlorophenyl)hydrazono]malononitrile (119e)

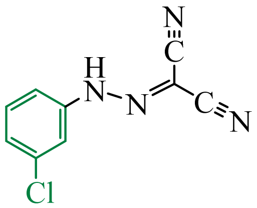

Yellow solid (14.66 g, 75%); m.p. 146–148 °C. 1H NMR: δ = 12.99 (br s, 1 H, NH), 7.50–7.38 (m, 3 H, H-2, H-5, H-6), 7.27–7.20 (m, 1 H, H-4). 13C NMR (125 MHz): δ = 142.7 (C-1), 133.9 (C-3), 131.2 (C-5), 125.3 (C-4), 116.1 (C-2), 115.0 (C-6), 113.9, 109.6 (2 × C≡N), 85.9 (C=N).

2-[(3-Fluorophenyl)hydrazono]malononitrile (119g)

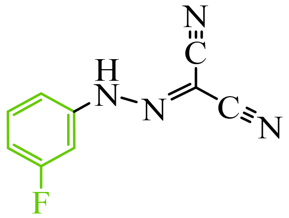

Yellow solid (13.55 g, 75%); mp 176–178 °C; 1H NMR: δ = 13.02 (br s, 1 H, NH), 7.48–7.40 (m, 1 H, H-5), 7.33–7.27 (m, 1 H, H-6), 7.26–7.21 (m, 1 H, H-2), 7.06–6.99 (m, 1 H, H-4). 13C NMR: δ = 162.57 (C-3, JC,F = 244.0 Hz), 143.1 (C-1, JC,F = 10.0 Hz), 131.3 (C-5, JC,F = 10.0 Hz), 113.9 (C≡N), 112.4 (C-6), 112.2 (C-4, JC,F = 21.0 Hz), 109.6 (C≡N), 103.5 (C-2, JC,F = 27.0 Hz), 85.8 (C=N).

12. Oxathiozolidinylpyrazoles [60]

Methyl 3-Cyano-4-(2-imino-4-oxothiazolidin-3-yl)-1-phenyl-1Hpyrazole-5-carboxylate (126a)

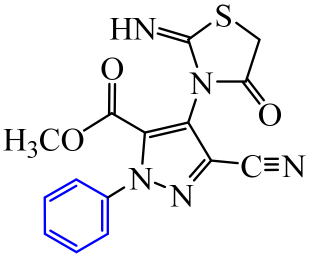

White solid (119 mg, 55%); mp 82–84 °C. IR (ATR): 3300 (NH), 2928, 2248 (C≡N), 1740 (estr C=O), 1633, 1619 (amide C=O, C=N), 1493, 1439, 1369, 1290, 1241, 1199, 1179, 1137, 1112, 1038, 1018 cm^{-1}. ^{1}H NMR: δ = 9.75 (s, 1 H, NH), 7.65–7.53 (m, 5 H, ArH), 4.38 (s, 2 H, H5″), 3.70 (s, 3 H, OCH3). ^{13}C NMR: δ = 170.4 (C-4″), 156.7 (CO2CH3), 155.8 (C-2″), 138.6 (C-1′), 130.8 (Cpyr), 130.1 (C-4′), 129.1 (2 C, C-3′, C-5′), 125.5 (2 C, C-2′, C-6′), 124.0 (Cpyr), 123.4 (Cpyr), 111.2 (C≡N), 53.0 (OCH3), 33.6 (C-5″). MS (ESI): m/z = 342 [M + H]+. HRMS (ESI): m/z [M – H]– calcd for C15H10N5O3S: 340.0510; found: 340.0503.

Methyl 1-(3-Methoxyphenyl)-3-cyano-4-(2-imino-4-oxothiazolidin-3-yl)-1H-pyrazole-5-carboxylate (126b)

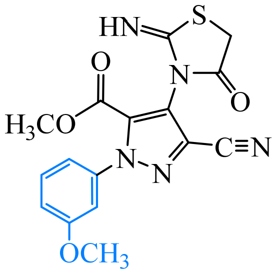

Yellow solid (60 mg, 70%); mp (dec.). IR (ATR): 3300 (NH), 2952, 2923, 2248 (C≡N), 1740 (ester C=O), 1631 (amide C=O, C=N), 1607, 1493, 1369, 1295, 1233, 1127, 1026 cm–1. 1H

NMR: δ = 9.74 (s, 1 H, NH), 7.49–7.43 (m, 1 H, H-5′), 7.26–7.22 (m, 1 H, H-2′), 7.19–7.12 (m, 2 H, H-4′, H-6′), 4.38 (s, 2 H, H-5″), 3.82 (s, 3 H, OCH3), 3.70 (s, 3 H, OCH3). 13C NMR: δ = 170.4 (C-4″), 159.5 (C-3′), 156.7 (CO2CH3), 155.8 (C-2″), 139.6 (C-1′), 130.9 (Cpyr), 129.9 (C-5′), 123.9 (Cpyr), 123.3 (Cpyr), 117.8 (C-6′), 116.2 (C-4′), 111.2 (2 C, C-2′, C≡N), 55.7 (OCH3), 53.0 (CO2CH3), 33.7 (C-5″). MS (ESI): m/z = 372 [M + H]+. HRMS (ESI): m/z [M + H]+ calcd for C16H14N5O4S: 372.0761; found: 372.0753.

Methyl 1-(3-Bromophenyl)-3-cyano-4-(2-imino-4-oxothiazolidin3-yl)-1H-pyrazole-5-carboxylate (126c)

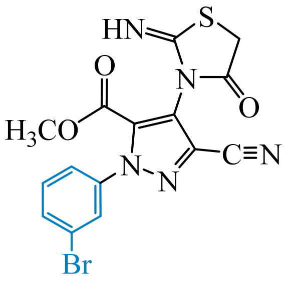

White solid (106 mg, 67%); mp 158–160 °C. IR (ATR): 3300 (NH), 2926, 2251 (C≡N), 1742 (ester C=O), 1619 (amide C=O, C=N), 1480, 1371, 1270, 1197, 1144, 1120, 1040 cm–1. 1H NMR: δ = 9.76 (s, 1 H, NH), 7.99 (t, J = 2.0 Hz, 1 H, H-2′), 7.80 (dd, J = 8.0, 2.0 Hz, 1 H, H-4′), 7.67 (dd, J = 7.5, 2.0 Hz, 1 H, H-6′), 7.52 (dd, J = 8.0, 7.5 Hz, 1 H, H-5′), 4.38 (s, 2 H, H-5″), 3.71 (s, 3 H, OCH3). 13C NMR: δ = 170.4 (C-4″), 156.6 (CO2CH3), 155.7 (C-2″), 139.7 (C-1′), 133.1 (C-4′), 130.9 (Cpyr), 130.8 (C-5′), 128.6 (C-2′), 125.1 (C-6′), 124.3 (Cpyr), 123.4 (Cpyr), 121.3 (C-3′), 111.1 (C≡N), 53.0 (OCH3), 33.7 (C-5″). MS (ESI): m/z = 420, 422 [M + H]+. HRMS (ESI): m/z [M – H]– calcd for C15H9 79BrN5O3S: 417.9615; found: 417.9623.

Methyl 1-(4-Bromophenyl)-3-cyano-4-(2-imino-4-oxothiazolidin3-yl)-1H-pyrazole-5-carboxylate (126d)

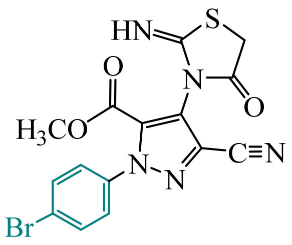

White solid (51 mg, 49%); m.p. 182–184 °C. IR (ATR): 3289 (NH), 2927, 2252 (C≡N), 1735 (ester C=O), 1616 (amide C=O, C=N), 1492, 1440, 1372, 1295, 1247, 1201, 1142, 1110, 1070, 1034, 1011 cm–1. 1H NMR: δ = 9.75 (s, 1 H, NH), 7.77 (d, J = 8.5 Hz, 2 H, H-3′, H-5′), 7.55 (d, J = 8.5 Hz, 2 H, H-2′, H-6′), 4.38 (s, 2 H, H-5″), 3.71 (s, 3 H, OCH3). 13C NMR: δ = 170.4

(C-4″), 156.7 (CO2CH3), 155.7 (C-2″), 137.8 (C-1′), 132.0 (2 C, C-2′, C-6′), 130.8 (Cpyr), 127.8 (2 C, C-3′, C-5′), 124.2 (Cq), 123.5 (Cq), 123.3 (Cq), 111.1 (C≡N), 53.0 (OCH3), 33.7 (C-5″). MS (ESI): m/z = 420, 422 [M + H]+.

Methyl 1-(3-Chlorophenyl)-3-cyano-4-(2-imino-4-oxothiazolidin3-yl)-1H-pyrazole-5-carboxylate (126e)

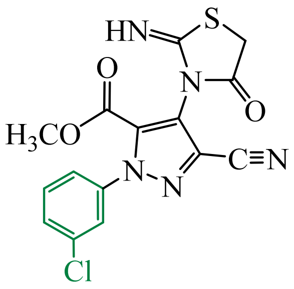

White solid (77 mg, 72%); m.p. (dec.). IR (ATR): 3320 (NH), 2248 (C≡N), 1721 (ester C=O), 1634 (amide C=O, C=N), 1587, 1552, 1481, 1347, 1310, 1240, 1130, 1043 cm–1. 1H NMR: δ = 9.76 (s, 1 H, NH), 7.91–7.86 (m, 1 H, H-2′), 7.71–7.56 (m, 3 H, H-4′, H-5′, H-6′), 4.37 (s, 2 H, H-5″), 3.71 (s, 3 H, CO2CH3). 13C NMR: δ = 170.4 (C-4″), 156.6 (CO2CH3), 155.7 (C-2″), 139.6 (C-1′), 133.2 (C-3′), 130.9 (Cpyr), 130.6 (CH), 130.2 (CH), 125.9 (C-2′), 124.7 (C-6′), 124.3 (Cpyr), 123.4 (Cpyr), 111.1 (C≡N), 53.0 (OCH3), 33.7 (C-5″). MS (ESI): m/z = 376 [M + H]+. HRMS (ESI): m/z [M + H]+ calcd for C15H1135ClN5O3S: 376.0266; found: 376.0261.

Methyl 1-(4-Chlorophenyl)-3-cyano-4-(2-imino-4-oxothiazolidin3-yl)-1H-pyrazole-5-carboxylate (126f)

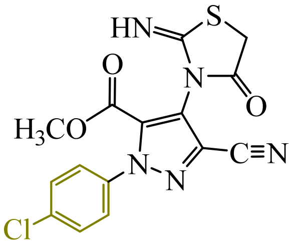

White solid (42 mg, 49%); m.p. 174–176 °C. IR (ATR): 3313 (NH), 2926, 2850, 2251 (C≡N), 1741 (ester C=O), 1619 (amide C=O, C=N), 1495, 1441, 1371, 1293, 1243, 1181, 1138, 1035, 1013 cm–1. 1H NMR: δ = 9.75 (s, 1 H, NH), 7.72–7.68 (m, 2 H, H-2′, H-6′), 7.66– 7.62 (m, 2 H, H-3′, H-5′), 4.38 (s, 2 H, H-5″), 3.71 (s, 3 H, OCH3). 13C NMR: δ = 170.4 (C-4″), 156.7 (CO2CH3), 155.7 (C-2″), 137.4 (C-1′), 134.7 (C-4′), 130.9 (Cpyr), 129.1 (2 C, C-2′, C-6′), 127.6 (2 C, C-3′, C-5′), 124.2 (Cpyr), 123.5 (Cpyr), 111.9 (C≡N), 53.0 (OCH3), 33.7 (C-5″). MS (ESI): m/z = 376 [M + H]+. HRMS (ESI): m/z [M + H]+ calcd for C15H1135ClN5O3S: 376.0266; found: 376.0261.

Methyl 1-(3-Fluorophenyl)-3-cyano-4-(2-imino-4-oxothiazolidin3-yl)-1H-pyrazole-5-carboxylate (126g)

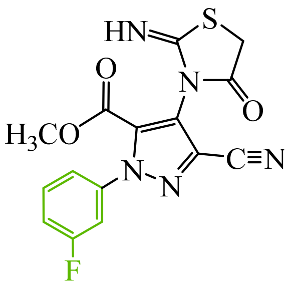

White solid (99 mg, 49%); mp 144–146 °C. IR (ATR): 3305 (NH), 3086, 2924, 2246 (C≡N), 1738 (ester C=O), 1633 (amide C=O, C=N), 1611, 1490, 1460, 1437, 1371, 1297, 1258, 1216, 1197, 1124, 1110, 1037, 903, 734 cm–1. 1H NMR: δ = 9.75 (s, 1 H, NH), 7.72–7.67 (m, 1 H, H-2′), 7.64–7.57 (m, 1 H, H-5′), 7.52–7.43 (m, 2 H, H-6′, H-4′), 4.37 (s, 2 H, H-5′′), 3.71 (s, 3 H, OCH3). 13C NMR: δ = 170.4 (C-4′′), 161.5 (d, JC,F = 246.0 Hz, C-3′), 156.6 (CO2CH3), 155.8 (C-2′′), 139.7 (d, JC,F = 11.0 Hz, C-1′), 131.0 (Cpyr), 130.8 (d, JC,F = 9.0 Hz, C-5′), 124.3 (Cpyr), 123.5 (Cpyr), 122.1 (C-6′), 117.2 (d, JC,F = 21.0 Hz, C-4′), 113.6 (d, JC,F = 26.5 Hz, C-2′), 111.7 (C≡N), 53.5 (OCH3), 33.7 (C-5′′). MS (ESI): m/z = 358 [M – H]–.

Methyl 1-(4-Fluorophenyl)-3-cyano-4-(2-imino-4-oxothiazolidin3-yl)-1H-pyrazole-5-carboxylate (126h)

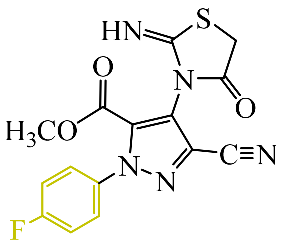

White solid (143 mg, 74%); mp 178–180 °C; IR (ATR): 3311 (NH), 2925, 2254 (C≡N), 1747 (ester C=O), 1614 (amide C=O, C=N), 1515, 1444, 1374, 1292, 1241, 1143, 1123 cm–1. 1H NMR: δ = 9.74 (s, 1 H, NH), 7.74–7.67 (m, 2 H, H-2′, H-6′), 7.43– 7.36 (m, 2 H, H-3′, H-5′), 4.38 (s, 2 H, H-5′′), 3.70 (s, 3 H, OCH3). 13C NMR: δ = 170.3 (C-5′′), 162.3 (d, JC,F = 248.0 Hz, C-4′), 156.5 (CO2CH3), 155.7 (C-2′′), 134.9 (C-1′), 130.8 (Cpyr), 128.1 (d, JC,F = 9.0 Hz, 2 C, C-2′, C-6′), 123.9 (Cpyr), 123.3 (Cpyr), 115.9 (d, JC,F = 23.5 Hz, 2 C, C3′, C-5′), 111.0 (C≡N), 52.9 (OCH3), 33.6 (C-5′′). MS (ESI): m/z = 358 [M – H]–. HRMS (ESI): m/z [M – H]– calcd for C15H9FN5O3S: 358.0416; found: 358.0407.

13. Triazenes [62]

2(4Methoxyphenyl)3methyl5(pyrrolidin1yl)2,3di hydro1,2,4triazine6carbonitrile (135a)

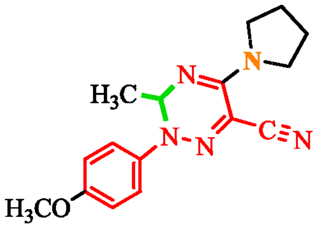

Yellow powder. Yield 0.12 g (40%), m.p. 107—108 °C. IR, ν/cm–1: 2205 (CN); 2860, 2880, 2960 (CH). 1H NMR (DMSOd6), δ: 1.24 (d, 3 H, Me, J = 6.0 Hz); 1.75—1.87 (m, 2 H, CH2); 1.89—2.00 (m, 2 H, CH2); 3.39—3.50 (m, 2 H, CH2); 3.51—3.57 (m, 2 H, CH2); 3.78 (s, 3 H, OMe); 5.96 (q, 1 H, C(3)H, J = 6.0 Hz); 7.00, 7.42 (both AA′XX′ system, 4 H, HAr, J = 9.1 Hz). 13C NMR (DMSOd6), δ: 17.6 (Me); 25.5, 48.5 (CH2); 55.9 (OMe); 68.3 (C(3)H); 110.5(CN); 115.2, 119.3 (CHAr); 117.6, 146.1 (C); 136.1, 157.6 (CAr). MS (EI, 70 eV), m/z (Irel (%)): 297 [M]+ (31). Found (%): C, 64.5; H, 6.5; N, 23.7. C16H19N5O. Calculated (%): C, 64.6; H, 6.4; N, 23.5.

2(4Methoxyphenyl)3methyl5(morpholin4yl)2,3di hydro1,2,4triazine6carbonitrile (135b)

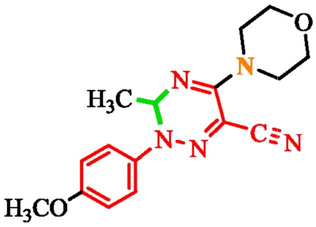

Yellow powder. Yield 0.22 g (70%), m.p. 101—102 °C. IR, ν/cm–1: 2205 (CN); 2855, 2880, 2920, 2960 (CH). 1H NMR (DMSOd6), δ: 1.23 (d, 3 H, Me, J = 6.0 Hz); 3.15—3.21 (m, 2 H, CH2); 3.38—3.44 (m, 2 H, CH2); 3.65—3.77 (m, 4 H, CH2); 3.81 (s, 3 H, OMe); 6.03 (q, 1 H, C(3)H, J = 6.0 Hz); 6.98, 7.42 (both AA′XX′ system, 4 H, HAr, J = 8.9 Hz). 13C NMR (DMSOd6), δ: 16.3 (Me); 47.9 (CH2); 55.4 (OMe); 65.6 (CH2); 68.1 (C(3)H); 110.6 (CN); 115.2, 119.6 (CHAr); 116.8, 150.3 (C); 135.9, 157.4 (CAr). MS (EI, 70 eV), m/z (Irel (%)): 313 [M]+ (23). Found (%): C, 61.3; H, 6.1; N, 22.4. C16H19N5O2. Calculated (%): C, 61.1; H, 6.3; N, 22.6.

2(4Methoxyphenyl)3phenyl5(pyrrolidin1yl)2,3di hydro1,2,4triazine6carbonitrile (135c)

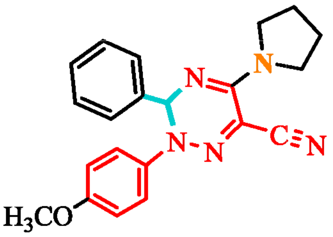

Yellow powder. Yield 0.29 g (81%), m.p. 101—102 °C. IR, ν/cm–1: 2210 (CN); 2880, 2940 (CH). 1H NMR (DMSOd6), δ: 1.80—1.87 (m, 2 H, CH2); 1.89—2.12 (m, 2 H, CH2); 3.39—3.50 (m, 2 H, CH2); 3.54—3.64 (m, 2 H, CH2); 3.78 (s, 3 H, OMe); 6.89 (s, 1 H, C(3)H); 6.92, 7.34 (both AA´XX´ system, 4 H, HAr, J = 9.1 Hz); 7.20—7.38 (m, 5 H, HAr). 13C NMR (DMSOd6), δ: 25.5, 48.6 (CH2); 55.9 (MeO); 73.3 (C(3)H); 110.7 (CN); 115.1, 119.5, 126.5, 128.6, 129.2 (CHAr); 117.3, 130.1, 139.3, 141.5, 145.2 (C). MS (EI, 70 eV), m/z (Irel (%)): 359 [M]+ (79). Found (%): C, 70.3; H, 5.6; N, 19.7. C21H21N5O. Calculated (%): C, 70.2; H, 5.9; N, 19.5.

2,3Diphenyl5(pyrrolidin1yl)2,3dihydro1,2,4triazine 6carbonitrile (135d)

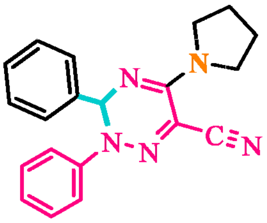

Yellow powder. Yield 0.27 g (81%), m.p. 98—99 °C. IR, ν/cm–1: 2210 (CN); 2840, 2875, 2950, 2980 (CH); 3060 (CHAr). 1H NMR (DMSOd6), δ: 1.72—1.86 (m, 2 H, CH2); 1.88—1.98 (m, 2 H, CH2); 3.42—3.50 (m, 2 H, CH2); 3.54—3.68 (m, 2 H, CH2); 6.92 (s, 1 H, C(3)H); 7.18—7.44 (m, 10 H, HAr). 13C NMR (DMSOd6), δ: 25.5, 48.4 (CH2); 73.2 (C(3)H); 113.7 (CN); 116.2 (C); 117.7, 126.1, 126.4, 128.9, 129.3, 130.0 (CHAr); 138.6, 143.0, 150.0 (C). MS (EI, 70 eV), m/z (Irel (%)): 329 [M]+ (35). Found (%): C, 72.6; H, 6.0; N, 21.6. C20H19N5. Calculated (%): C, 72.9; H, 5.8; N, 21.3.

2(4Chlorophenyl)3phenyl5(pyrrolidin1yl)2,3dihydro 1,2,4triazine6carbonitrile (135e)

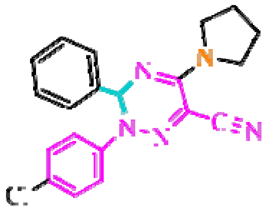

Green powder. Yield 0.24 g (67%), m.p. 105—106 °C. IR, ν/cm–1: 2210 (CN); 2860, 2880 (CH). 1H NMR (DMSOd6), δ: 1.86—1.89 (m, 2 H, CH2); 1.90—2.02 (m, 2 H, CH2); 3.48—

3.58 (m, 2 H, CH2); 3.60—3.69 (m, 2 H, CH2); 6.97 (s, 1 H, C(3)H); 7.20 (d, 2 H, HAr, J = 8.0 Hz); 7.29—7.33 (m, 3 H, HAr); 7.38—7.46 (m, 4 H, HAr). 13C NMR (DMSOd6), δ: 25.5, 48.7 (CH2); 72.5 (C(3)H); 112.4 (CN); 116.7 (C); 119.3, 126.5, 128.5, 129.3, 129.9 (CHAr); 130.1, 139.3, 141.5, 145.2 (C). MS (EI, 70 eV), m/z (Irel (%)): 363 [M]+ (14). Found (%): C, 66.0; H, 5.0; N, 19.2. C20H18N5Cl. Calculat ed (%): C, 66.2; H, 4.8; N, 19.4.

2(4Nitrophenyl)3phenyl5(pyrrolidin1yl)2,3dihydro 1,2,4triazine6carbonitrile (7f)

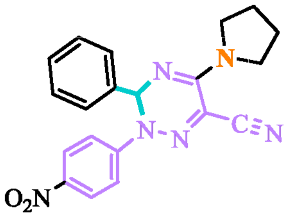

Redbrown powder. Yield 0.27 g (73%), m.p. 111—112 °C. IR, ν/cm–1: 2220 (CN); 2860, 2945, 2970 (CH), 3070, 3115 (CHAr). 1H NMR (DMSOd6), δ: 1.80—1.90 (m, 2 H, CH2); 1.91—2.15 (m, 2 H, CH2); 3.50—3.56 (m, 2 H, CH2); 3.57—3.68 (m, 2 H, CH2); 7.16 (s, 1 H, C(3)H); 7.22—7.41 (m, 5 H, HAr); 7.67, 8.24 (both AA′XX′ system, 4 H, HAr, J = 9.1 Hz). 13C NMR (DMSOd6), δ: 25.5, 48.7 (CH2); 72.2 (C(3)H); 115.4 (CN); 116.2 (C); 117.3, 125.9, 126.4, 128.9, 129.4 (CHAr); 139.2, 144.0, 144.5, 147.7 (C). MS (EI, 70 eV), m/z (Irel (%)): 374 [M]+ (21). Found (%): C, 64.0; H, 5.0; N, 22.7. C20H18N6O2. Calculated (%): C, 64.2; H, 4.9; N, 22.4.

2,3Diphenyl5(piperidin1yl)2,3dihydro1,2,4triazine 6carbonitrile (135g)

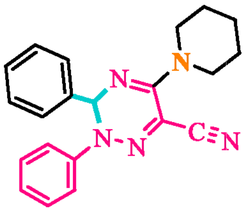

Yellow powder. Yield 0.27 g (80%), m.p. 104—105 °C. IR, ν/cm–1: 2215 (CN); 2850, 2935 (CH). 1H NMR (DMSOd6), δ: 1.51—1.65 (m, 6 H, CH2); 3.20—3.26 (m, 2 H, CH2); 3.69—3.94 (m, 2 H, CH2); 7.20 (s, 1 H, C(3)H); 7.21—7.50 (m, 10 H, HAr). 13C NMR (DMSOd6), δ: 24.1, 25.5, 49.0 (CH2); 72.7 (C(3)H); 113.7 (CN); 116.2, 143.0, (C); 117.7, 126.0, 126.4, 128.9, 129.3, 130.0 (CHAr); 138.6, 150.8 (CAr). MS (EI, 70 eV), m/z (Irel (%)): 343 [M]+ (37). Found (%): C, 73.3; H, 5.9; N, 20.7. C21H21N5. Calculated (%): C, 73.4; H, 6.2; N, 20.4.

5Methylamino2(4nitrophenyl)3phenyl2,3dihydro 1,2,4triazine6carbonitrile (135i)

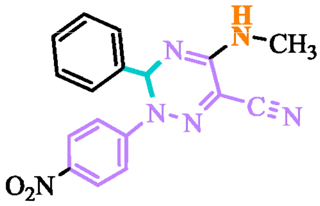

Redyellow powder. Yield 0.17 g (52%), m.p. 184—185 °C. IR, ν/cm–1: 2200 (CN); 2815 (CH); 3080, 3110 (CHAr); 3400 (NH). 1H NMR (DMSOd6), δ: 2.68 (d, 3 H, NHMe, J = 4.5 Hz); 5.08 (q, 1 H, NHMe, J = 4.5 Hz); 7.16 (s, 1 H, C(3)H); 7.27—7.38 (m, 5 H, HAr); 7.65, 8.24 (both AA´XX´ system, 4 H, HAr, J = 9.3 Hz). 13C NMR (DMSOd6), δ: 27.5 (Me); 71.5 (C(3)H); 113.8 (CN); 116.8, 125.3, 125.8, 128.4, 129.0 (CHAr); 139.5, 143.3, 143.4, 143.7, 147.5 (C). MS (EI, 70 eV), m/z (Irel (%)): 334 [M]+ (57). Found (%): C, 61.2; H, 4.6; N, 25.0. C17H14N6O2. Calculated (%): C, 61.1; H, 4.3; N, 25.1.

14. Pyrimidines [63]

(E)-5-(phenyldiazenyl)pyrimidine-2,4,6-triamine (139a)

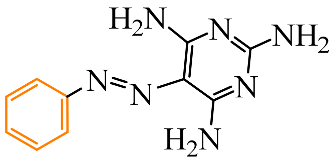

Pale brown crystals from ethanol, yield 53%, 0.607 g, m.p. 171-173ºC. IR (KBr): υ/cm-1 = 3441-3286 (3NH2), 3048 (CH aromatic), 1656 (C=N). 1 H NMR (DMSO-d6) δ = 4.22, 4.63, 4.91 (3s, 6H, D2O-exchangeable, 3NH2), 6.87-7.23 (m, 5H, C6H5). 13C NMR: δ = 122.3, 125.4, 126.8, 129.2, 131.1, 134.1, 135.3, 137.2, 138.8, 140.5 (pyrimidine C, C6H5 C). Calcd for C10H11N7 (229.24): C, 52.39; H, 4.84; N, 42.77%. Found: C, 52.64; H, 4.57; N, 42.99%.

(E)-5-((4-chlorophenyl)diazenyl)pyrimidine-2,4,6-triamine (139b)

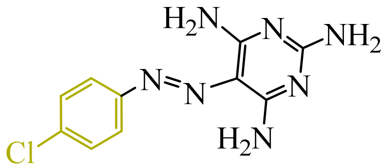

Off white crystals from ethanol, yield 48%, 0.633 g, m.p. 152-154ºC. IR (KBr): υ/cm-1 = 3467-3321 (3NH2), 3054 (CH aromatic), 1652 (C=N). 1 H NMR (DMSO-d6) δ = 4.43, 4.68, 5.23 (3s, 6H, D2O-exchangeable, 3NH2), 7.25-7.38 (d.d, 4H, C6H4). 13C NMR: δ = 120.2, 123.8, 126.2, 129.4, 132.5, 134.7, 136.2, 139.7, 140.8, 141.9, 143.1 (pyrimidine C, C6H4 C). Calcd for C10H10ClN7 (263.69): C, 45.55; H, 3.82; N, 37.18%. Found: C, 45.31; H, 3.56; N, 37.44%.

15. 2,2-Dicyano-3-methyl-3-vinyl-N-phenyla~etidin-4-one (143) [67]

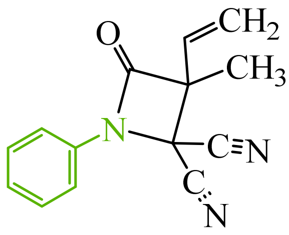

Oil; IR (film) 2255 (vw), 1790,1600,1500 cm-'; 'H NMR (CDC13) 6 1.87 (s, 3 H, Me), 5.55-5.82 (m, 2 H, of HC=CH,), 5.97-6.30 (m, 1 H of HC=CH2), 7.23-7.65 (m, 5 H, arom); '% NMR (CDCl,) 6 18.11 (Me), 52.53 (C), 69.54 (C), 110.65 (2 C), 117.20 (2 CH), 122.60 (CH,), 126.69 (CH), 129.83 (2 CH), 130.3 (CH), 134.24 (C), 163.14 (C); mass spectrum, m/e 237 (M'), 119,82. Anal. Calcd for C1,Hl1N3O: C, 70.87; H, 4.67; N, 17.71. Found: C, 70.98; H, 4.73; N, 17.65.

16. 6,6-Dicyano-4-(mesitylimino)-5-phenyl-5-azaspiro[2.3]hexane (145) [68]

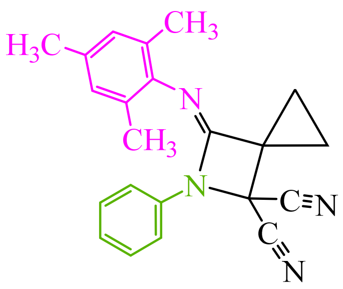

m.p. 203-206 "C (from ethyl ether); IR (CC14, C2C14, CS2) 2255 vw C=N), 1730 (NC=N) cm-'; 'H NMR (CDClJ 6 1.03-1.23 (m, 2 H), 1.23-1.53 (m, 2 H), 2.15 (s, 6 H, 2 ortho CH,), 2.23 (s, 3 H, 2 ortho CH,), 6.73-6.77 (br, 2 H, a), 7.1-7.80 (m, 5 H, a); 13C NMR (CDClJ 6 11.59 (2 CH3), (2 C of C=N), 116.43 (2 CH, a), 124.87 (CH, a), 127.78 (2 C, a), 18.36 (2 CH3), 20.70 (1 CH3), 42.59 (C3), 51.58 (C Of C(CN)J, 111.46 128.52 (2 CH, a), 129.69 (2 CH, a), 133.21 (C, a), 136.86 (C, a), 139.29 (C, a), 151.06 (C of NC=N); mass spectrum, m/e 340 (M'), 325, 236, 185, 104. Anal. Calcd for Cz2H20N4: C, 77.62; H, 5.92; N, 16.46. Found: C, 78.01; H, 5.87; N, 16.51.

17. 2,3-Bis(4-aminophenyl)buta-1,3-diene-1,1,4,4-tetracarbonitrile (156) [72]

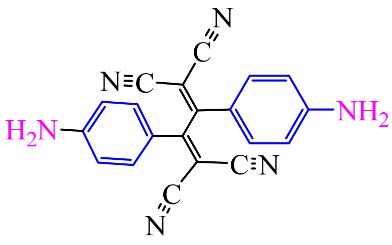

0.26 g (78%). 1H-NMR (500 MHz, methanol-d4) δ ppm 7.69 (d, J = 10 Hz, 2H), 6.68 (d, J = 10 Hz, 2H), 4.60 (s, 1H), 13C-NMR (125 MHz, methanol-d4) δ ppm 167.5, 157.9, 134.6,

120.1, 116.2, 115.4, 74.8. HR-MS (MALDI-TOF) calc. mass for C20H12N6 [M+Na]+ = 359.10211, found 359.10038.

High-Pressure Liquid Chromatography (HPLC) Data

(E)-3-(5-((2-cyanoquinazolin-4-yl)(methyl)amino)-2- methoxyphenyl)-N-hydroxyacrylamide (62) [30]

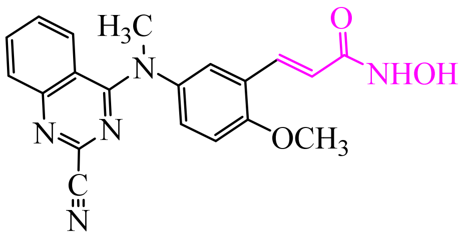

The purity of the final compounds was determined to be ≥ 95% using high-pressure liquid chromatography-mass spectrometry (HPLC-MS) on a Waters Alliance 2695 (HPLC) and LCT Premier.

HPLC (ACN/Water, 1/9 to 10/0 in 10 min): 100%
